# Supplementary figures and images for: Prior expectations evoke stimulus-specific activity in the deep layers of the primary visual cortex
Source: PLoS Biol. 2020 Dec 7;18(12):e3001023. doi: 10.1371/journal.pbio.3001023 (PMC7746273; doi:10.1371/journal.pbio.3001023)

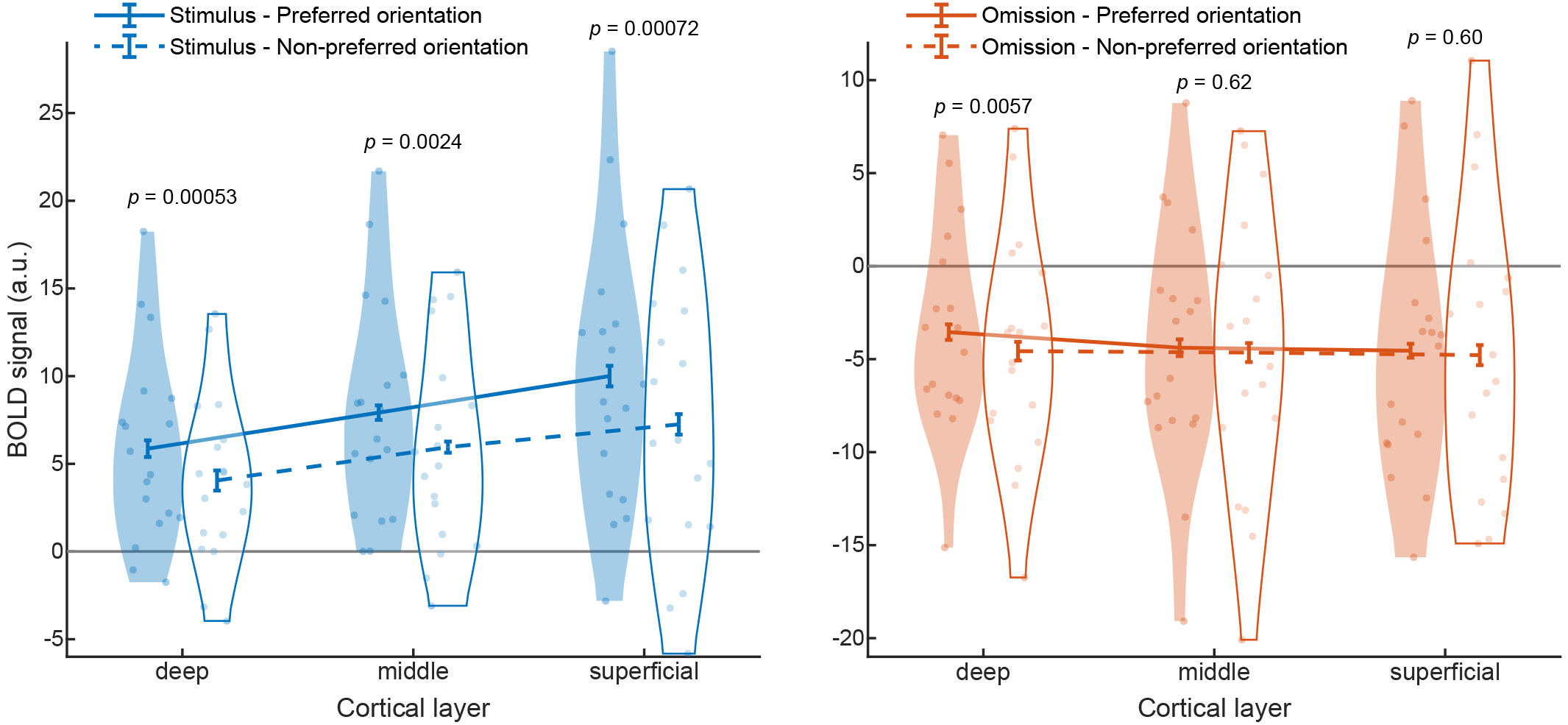

Supplement: S1 Fig — BOLD responses are higher in subpopulations preferring the (expected) orientation in all layers for presented stimuli and deep layers only for expected-but-omitted stimuli. Note that the omission responses are overall negative. This is likely the result of the fact that the current study employed a fast event-related design without an explicit baseline period. Specifically, in this type of design, the baseline is effectively the mean signal, and when a stimulus is omitted, during a run in which stimuli are presented most of the time, the signal in V1 is likely to be lower than average. Essentially, this type of design is optimal for detecting differences between conditions (stimulus vs. omission or 45° stimulus/omission vs. 135° stimulus/omission), which was our main interest here, but suboptimal for detecting main effects of single conditions (e.g., stimulus vs. baseline or omission vs. baseline). Dots represent individual participants, and curved shapes indicate density. Error bars indicate within-subject SEM. Data are available at osf.io/k54p3. BOLD, blood oxygen level–dependent; SEM, standard error of the mean; V1, primary visual cortex. (TIF) [file pbio.3001023.s001.tif]

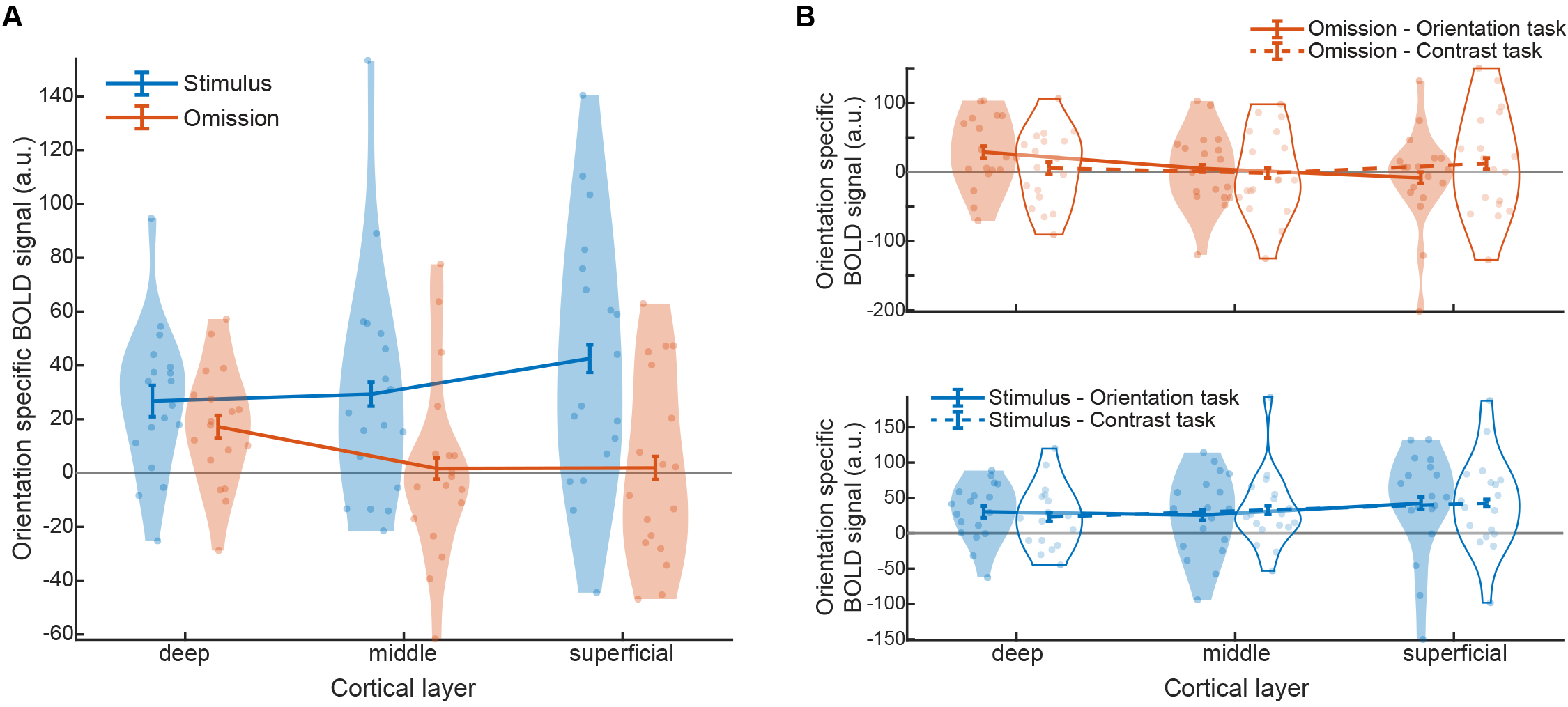

Supplement: S2 Fig — Normalising and weighting voxel time courses by orientation selectivity was omitted in this control analysis. (A) Orientation-specific BOLD response to presented (blue) and expected-but-omitted (orange) gratings in the different layers of V1, averaged over tasks. (B) Orientation-specific BOLD response to expected-but-omitted (orange, top panel) and presented (blue, bottom panel) gratings, separately for the orientation (solid lines, filled shapes) and contrast (dashed lines, open shapes) tasks. Dots represent individual participants, and curved shapes indicate density. Error bars indicate within-subject SEM. Data are available at osf.io/k54p3. BOLD, blood oxygen level–dependent; SEM, standard error of the mean; V1, primary visual cortex. (TIF) [file pbio.3001023.s002.tif]

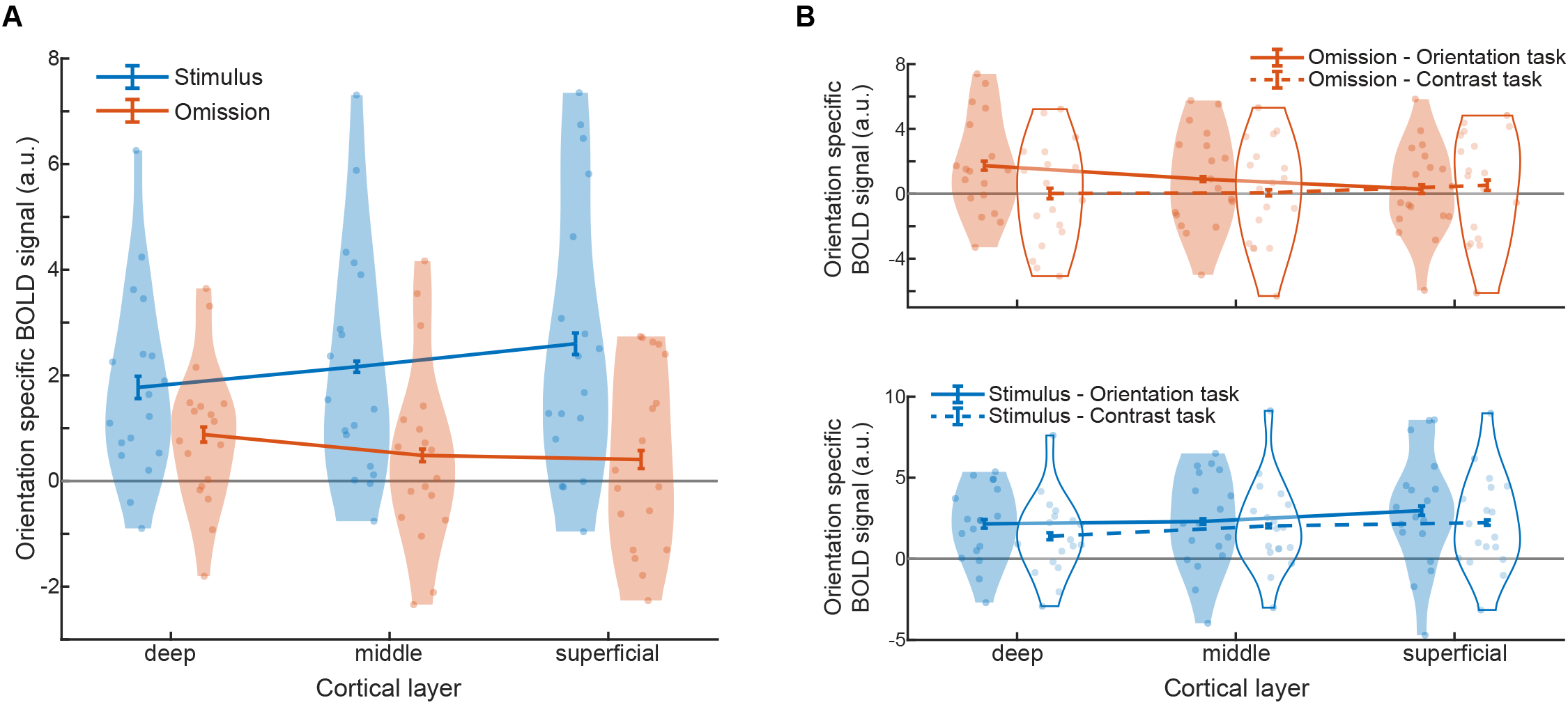

Supplement: S3 Fig — (A) Orientation-specific BOLD response to presented (blue) and expected-but-omitted (orange) gratings in the different layers of V1, averaged over tasks. (B) Orientation-specific BOLD response to expected-but-omitted (orange, top panel) and presented (blue, bottom panel) gratings, separately for the orientation (solid lines, filled shapes) and contrast (dashed lines, open shapes) tasks. Dots represent individual participants, and curved shapes indicate density. Error bars indicate within-subject SEM. Data are available at osf.io/k54p3. BOLD, blood oxygen level–dependent; GLM, general linear model; SEM, standard error of the mean; V1, primary visual cortex. (TIF) [file pbio.3001023.s003.tif]

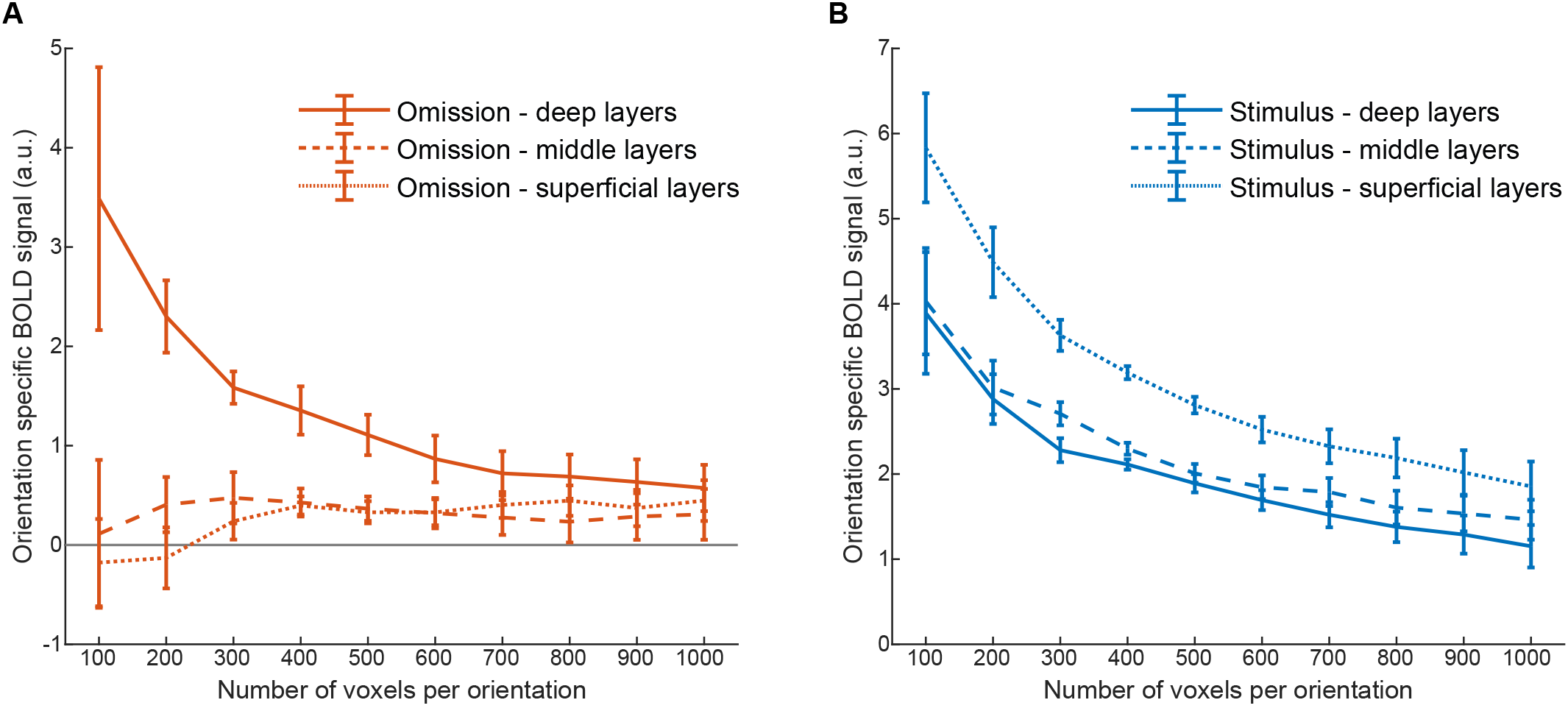

Supplement: S4 Fig — (A) Orientation-specific BOLD response to expected-but-omitted gratings in the different layers of V1, averaged over tasks. (B) Orientation-specific BOLD response to presented gratings in the different layers of V1, averaged over tasks. Error bars indicate within-subject SEM. Data are available at osf.io/k54p3. BOLD, blood oxygen level–dependent; SEM, standard error of the mean; V1, primary visual cortex. (TIF) [file pbio.3001023.s004.tif]

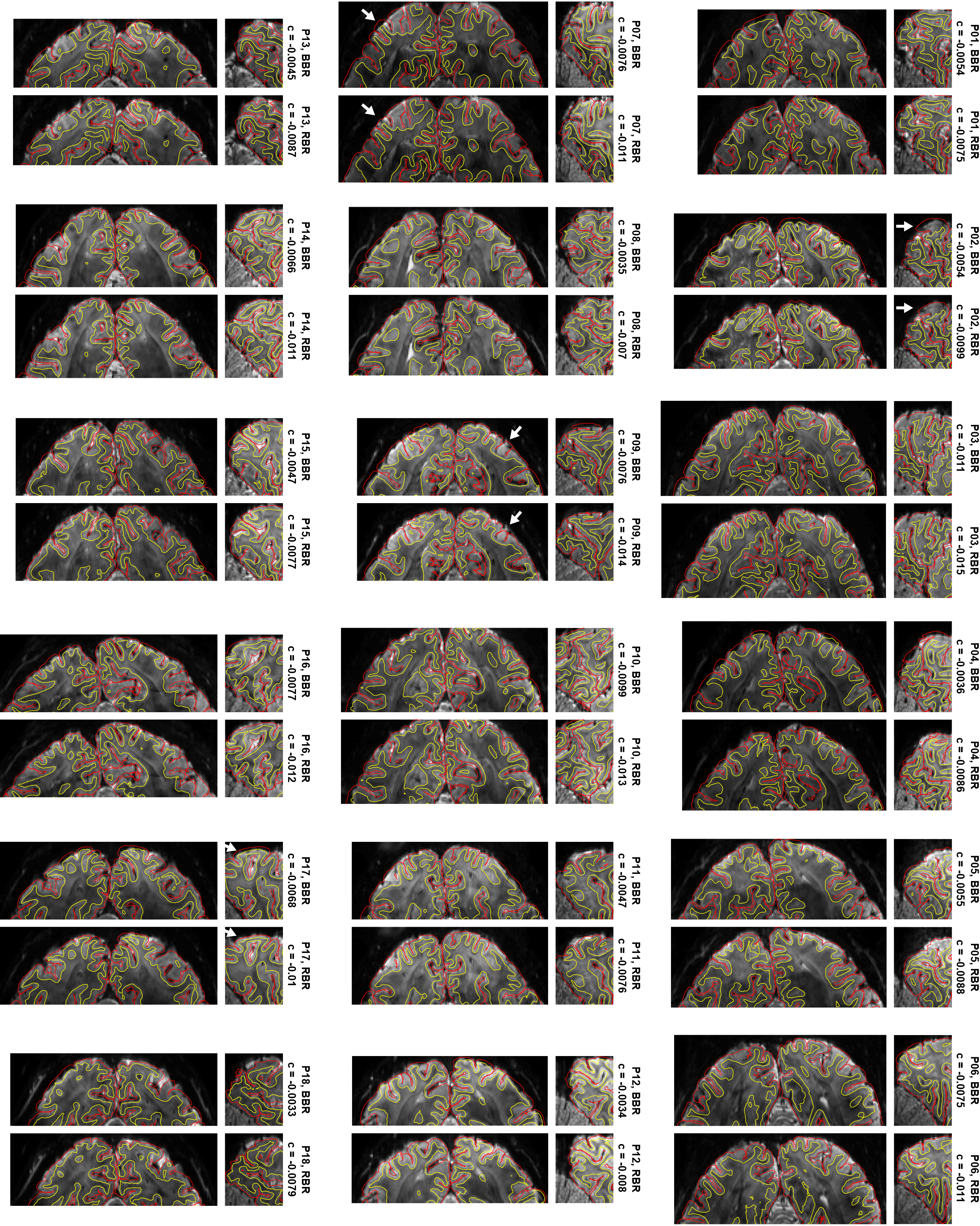

Supplement: S5 Fig — Registrations are shown after rigid-body registration only (BBR), as well as after RBR. RBR increased absolute GM–WM contrast (c) in all participants. Arrows highlight locations where RBR improved registration. BBR, boundary-based registration; EPI, echo planar imaging; GM, grey matter; RBR, recursive boundary registration; WM, white matter. (TIF) [file pbio.3001023.s005.tif]

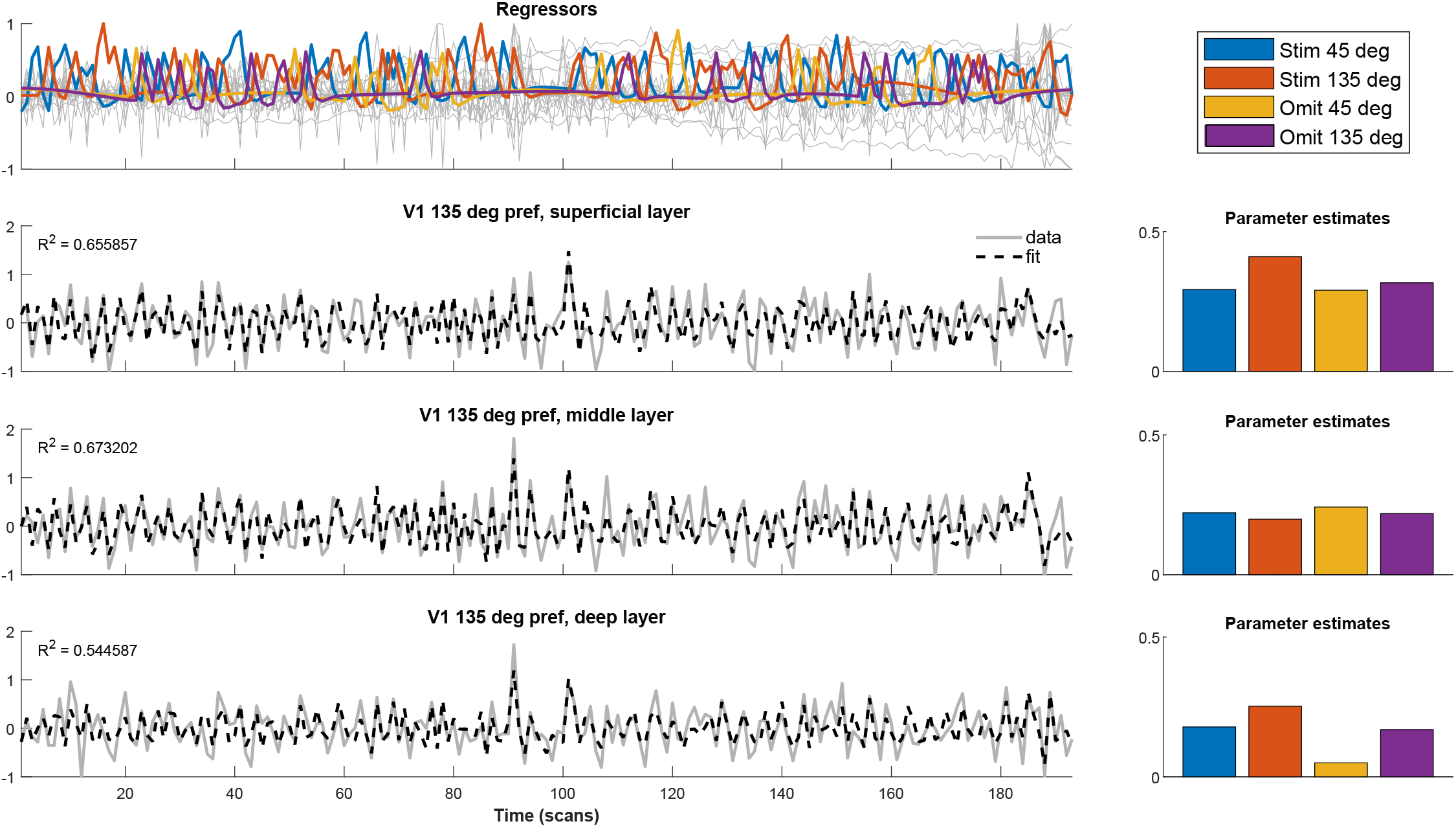

Supplement: S6 Fig — Example model and data shown for 1 participant (P1) and 1 ROI (V1, 135 degree preferring voxels). Left, top panel: regressors used in the temporal GLM. Coloured time courses indicate regressors for the 4 conditions of interest, and grey time courses indicate nuisance regressors (i.e., head motion). Left, 3 bottom panels: fMRI time courses in each of the 3 GM layers (solid grey) and time courses fit by GLM (dashed black). Right, 3 bottom panels: parameter estimates for the 4 regressors of interest, quantifying the amplitude of the BOLD response evoked by the 4 conditions. These parameter estimates constitute the main results as shown in Fig 3. Data are available at osf.io/k54p3. BOLD, blood oxygen level–dependent; fMRI, functional magnetic resonance imaging; GLM, general linear model; GM, grey matter; ROI, region of interest. (TIF) [file pbio.3001023.s006.tif]
