# Supplementary material for: Prior expectations evoke stimulus-specific activity in the deep layers of the primary visual cortex
Source: PLoS Biol. 2020 Dec 7;18(12):e3001023. doi: 10.1371/journal.pbio.3001023 (PMC7746273; doi:10.1371/journal.pbio.3001023)
Supplement: Attachment — Submitted filename: Response_to_reviewers.pdf [file pbio.3001023.s007.pdf]

We would like to thank the reviewers for their thorough and constructive assessment of our manuscript. We have revised the manuscript to address their points of critique, which we believe has led to a greatly improved paper. Our previous manuscript was conceived as a short report, however the reviewers helped us realise that the paper would benefit from presenting a lot more detail on the analysis and data quality. Our point-by-point responses are detailed below.

Reviewer 1

1. What was the echo spacing and total read-out time of the functional volumes? This information is needed in order to estimate the amount of distortion present in the functional data, which is of particular importance given that distortion compensation was not performed.

The echo spacing was 1.27 ms. The matrix size in the phase-encoding (PE) direction was 240, the acceleration factor in the PE direction was 4, and the Partial Fourier factor was 6/8. The effective EPI readout time (which dictates the amount of distortions) was therefore 1.27*240/4 = 76.2ms. We apologise for not providing the echo spacing previously, and how included it in the Materials and Methods.

We would like to emphasise that we do compensate for distortions, but performing a novel method known as Recursive Boundary Registration (RBR) (Van Mourik et al., 2019). This method consists of applying Boundary Based Registration (BBR) on progressively smaller subregions of the brain, in order to correct for local distortions. Importantly, these local registrations allow scaling in the PE direction. In other words, we compensate for distortions not by unwarping the EPI images, but by non-linearly registering the cortical boundaries to the distorted EPI images. This method has been used successfully in previous layer-specific fMRI studies (Kok et al., 2016; Scheeringa et al., 2016; Lawrence et al., 2018, 2019). We discuss this approach, and its effectiveness, in more detail in response to point 3.

2. How was the temporal stability of the pre-processed functional data ascertained? The motion-based exclusion criteria, while described as "strict" in the manuscript, seem to allow for an unlimited amount of motion from the beginning of the scanning session to the end, as long as motion transients were smaller than 1 mm. Image distortions change as head position changes, and the shape-change of the brain during the course of the scanning session could frustrate even the best motion compensation algorithm. Thus, there are no guarantees that a given voxel subjected to GLM analyses contains the same tissue in the final volume that it did in the first volume. In the best case, this causes only blurring. In the worst case, however, the image to which anatomical surfaces was warped could easily fail to represent the location of tissue during the bulk of the scanning session. Since large amounts of motion were possibly allowed and distortion compensation was not performed, the manuscript needs to include details about how the authors were sure that their images were stable after pre-processing.

We thank the reviewer for raising this important point. There are indeed two orthogonal problems with head motion in high-field fMRI: noise introduced into the MR signal by motion transients, and differences in image distortion as a function of large head movements within or between runs. Our head motion criteria we reported in the manuscript only focused on the former: volume-to-volume transients. To address this, we have now also quantified the maximum change in head position across the session for each participant. For all participants, the maximum change in head position in any direction was within 4 mm (1.9 +/- 0.8 mm, mean +/- SD over participants) of the mean head

position (to which the anatomical boundaries were registered). Changes in head position of this magnitude are unlikely to cause distortions to the extent that motion compensation would be significantly impaired.

In order to quantify the temporal stability of the EPI signal, we calculated the mean tSNR (defined as mean signal / SD over time) for in-brain voxels. We found that tSNR was significantly higher after (12.5 +/- 1.6, mean +/- SD over participants) than before (9.2 +/- 1.6) spatial realignment of the EPI volumes, demonstrating the benefit of our motion compensation approach.

We now report these data in the Materials and Methods section.

3. How was the accuracy of cortical depth assignment for functional voxels ascertained? The manuscript needs to include details about the accuracy of the warping of the anatomical boundaries to the functional data. BBR is an excellent algorithm when it has adequate image contrast to work with, but the GM/WM boundary is notoriously devoid of contrast in most functional acquisitions - this is evident in the small functional section shown in Fig. 1. It is likely that warped anatomical surfaces were able to find the GM/pial boundary on the outer surface of the brain, but it seems unlikely that, without guidance by some kind of additional dataset acquired during the scanning session to enhance GM/WM contrast, RBR would be able to reliably fit the distorted functional data. To conform with best practices for reporting laminar analyses, the authors need to show examples of functional/anatomical registration for multiple subjects or provide an independent metric that proves that functional GM was correctly identified.

As mentioned in response to point 1, we used a method known as Recursive Boundary Registration (RBR), that corrects distortions between an anatomical and an EPI volume by recursively applying Boundary Based Registration (BBR) on progressively smaller subregions of the brain, based on the grey-white matter contrast (Van Mourik et al., 2019).

While a functional image is too low in contrast for generating a cortical reconstruction, it can be used in this registration procedure. We and others have found the contrast to be sufficient to optimise the average contrast across the boundary in order to achieve a better realignment. In order to register the anatomical and functional volumes, the constructed surface was moved to the mean functional image. The average contrast in the functional volume across the grey-white matter boundary was computed and used as a cost function to optimise the transformation parameters of the registration.

At each iteration of the RBR, the surface meshes were split into two, and BBR (with 7 degrees of freedom: rotation and translation along all three dimensions, and scaling along the phase-encoding direction) was performed on each partition separately. Here, we ran six such iterations. The splits were made along the cardinal axes of the volume, such that the number of vertices was equal for both parts. The plane for the second cut is orthogonal to the first, the third orthogonal to the first two. The median displacement was taken after running the recursive algorithm six times, in which different splitting orders where used, comprised of all six permutations of x, y and z.

This recursive boundary registration, performed subsequent to a ‘standard’ whole brain rigid-body BBR, increased the average contrast across the gray-white matter boundary from -0.0060 (+/- 0.0022, mean +/- SD) to -0.0100 (+/- 0.0024, mean +/- SD), an increase of 75%. (Note that the contrast values are negative since white matter is darker than gray matter on T2* images.)

We now provide visualisations and quantifications of the registration (both pre- and post-RBR) in individual participants in the new Figure S1, reproduced below for convenience.

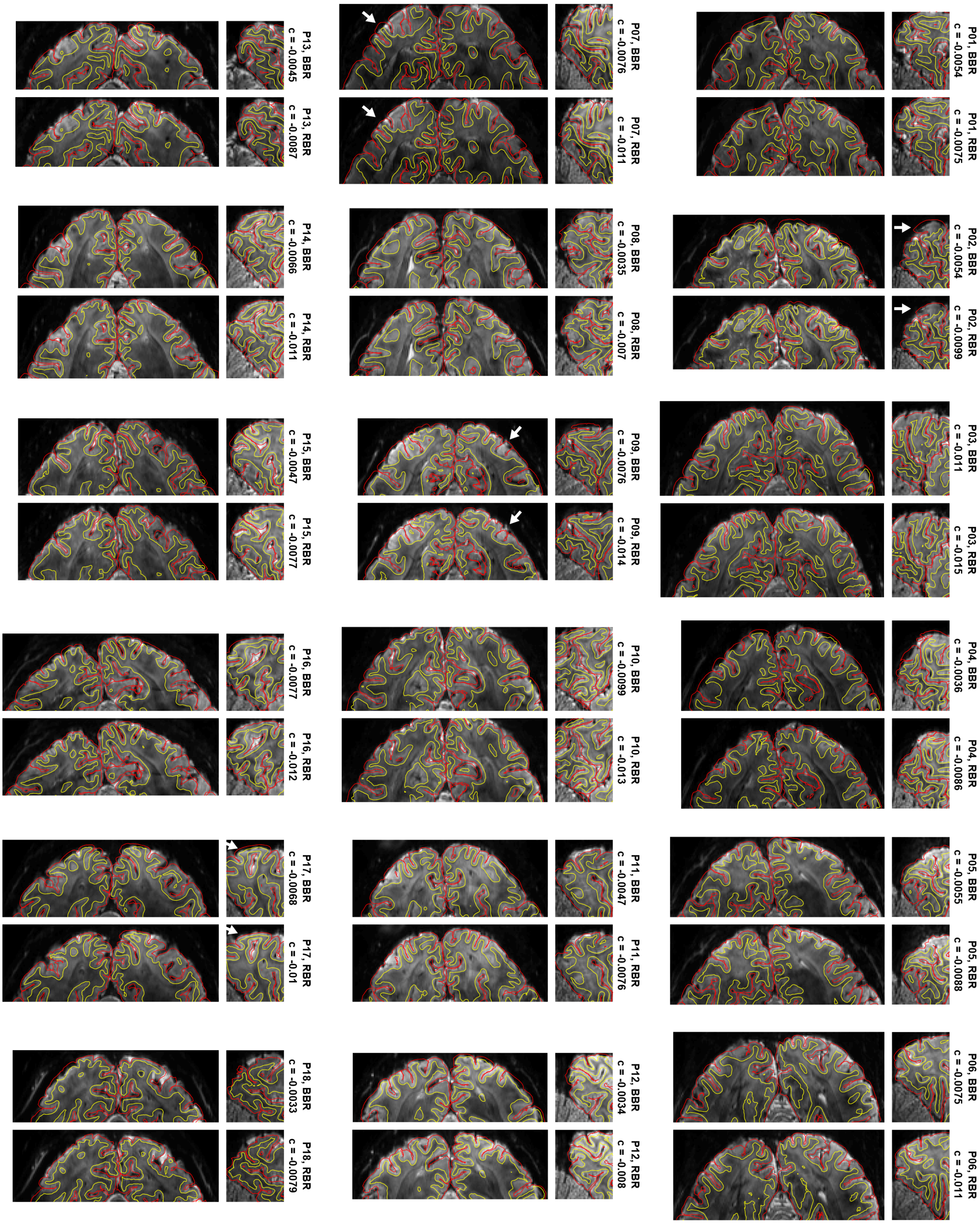

Fig. S1. Registration of cortical boundaries to mean EPI for all participants. Registrations are shown after rigid-body registration only (BBR), as well as after RBR. RBR increased absolute gray-white matter contrast (c) in all participants. Arrows highlight locations where RBR improved registration.

Finally, in order to confirm that the method correctly identified gray matter, we quantified the raw signal in the EPI volumes for each of the three gray matter layers, as well as white matter and CSF. Note that these results were obtained from the exact same data as our main results, by simply averaging the EPI time courses extracted from our ROIs (V1 45 degree preferring voxels and V1 135 preferring voxels, respectively, averaged together for this purpose) by the layer-specific spatial GLM (Figure R1). As expected, the signal intensity was higher in the three GM layers than in WM (t_{17} = 5.17, p = 7.7 x 10^{-5}) and outside of the brain (t_{17} = 3.81, p = 0.0014). We now report this quantification in the manuscript.

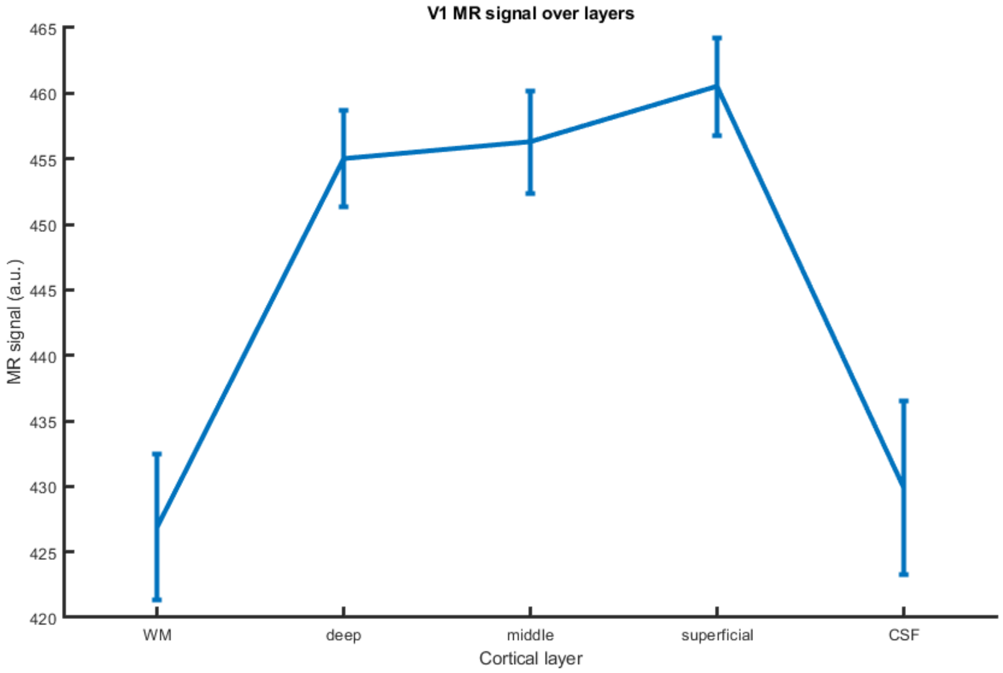

Figure R1. Raw MR signal intensity from EPI data, across layers. Error bars indicate within-participants SEM.

4. What kind of laminar distribution did the V1 ROIs have? The ROI-definition methods are decidedly atypical, picking 500 + 500 highest-contrast voxels. In an analysis of V1 response to large gratings, one expects 10's of thousands of active voxels. Picking such a small subset seems like it would leave the analysis vulnerable to domination by spatially non-selective signals from large veins and other biases. Even if large surface veins aren't at play, it is, for example, possible that the vast majority of the orientation-preferring voxels were in deep layers, such that the analysis only had enough power to detect orientation preferences in deep layers. I'm not saying that I think this is the case; I'm just saying that the manuscript cannot be evaluated without information about the distribution in space and depth (e.g., in the form of an image showing representative ROIs, plus laminar profiles of voxel counts and some kind of statement about the spatial distribution of selected voxels and which portion of the stimuli they likely represented).

The ROI definitions we used were identical to (indeed, based on) previous studies that successfully resolved orientation-specific BOLD signals with layer specificity (Lawrence et al., 2018, 2019). In fact, we matched our analysis approach as closely as possible to those previous papers, to be able to compare our effects of prior expectations on orientation-specific BOLD signals to the effects of

working memory (Lawrence et al. 2018) and attention (Lawrence et al. 2019) as well as possible. We now make this logic explicit in our manuscript (p. 19).

As the reviewer says, the first step, selecting V1 voxels that respond significantly ($t > 2.3$, $p < 0.05$) to the large gratings in our functional localiser, yielded thousands of active voxels per participant (6009 +/- 2033, mean +/- SD). In order to obtain a reliable orientation-specific BOLD signal, from this large set of voxels we selected the 500 voxels with the most reliable (largest absolute T value) for 45 > 135 degree gratings, and vice versa. This was done because most voxels will not have a reliable preference, but respond equally to both orientations. Thus, the selected voxels are a subset of those that respond to the large gratings, i.e., those that have a population receptive field on the region of the visual field covered by the gratings (i.e., the central 10 degrees, absent the central 1 degree). In terms of anatomical location, these voxels are located in the posterior occipital cortex. Since we did not map the population receptive fields of individual voxels, the exact distribution of the selected voxels' receptive fields over the visual field is not known.

We now show images of representative ROIs in a new Figure 2, reproduced below for convenience.

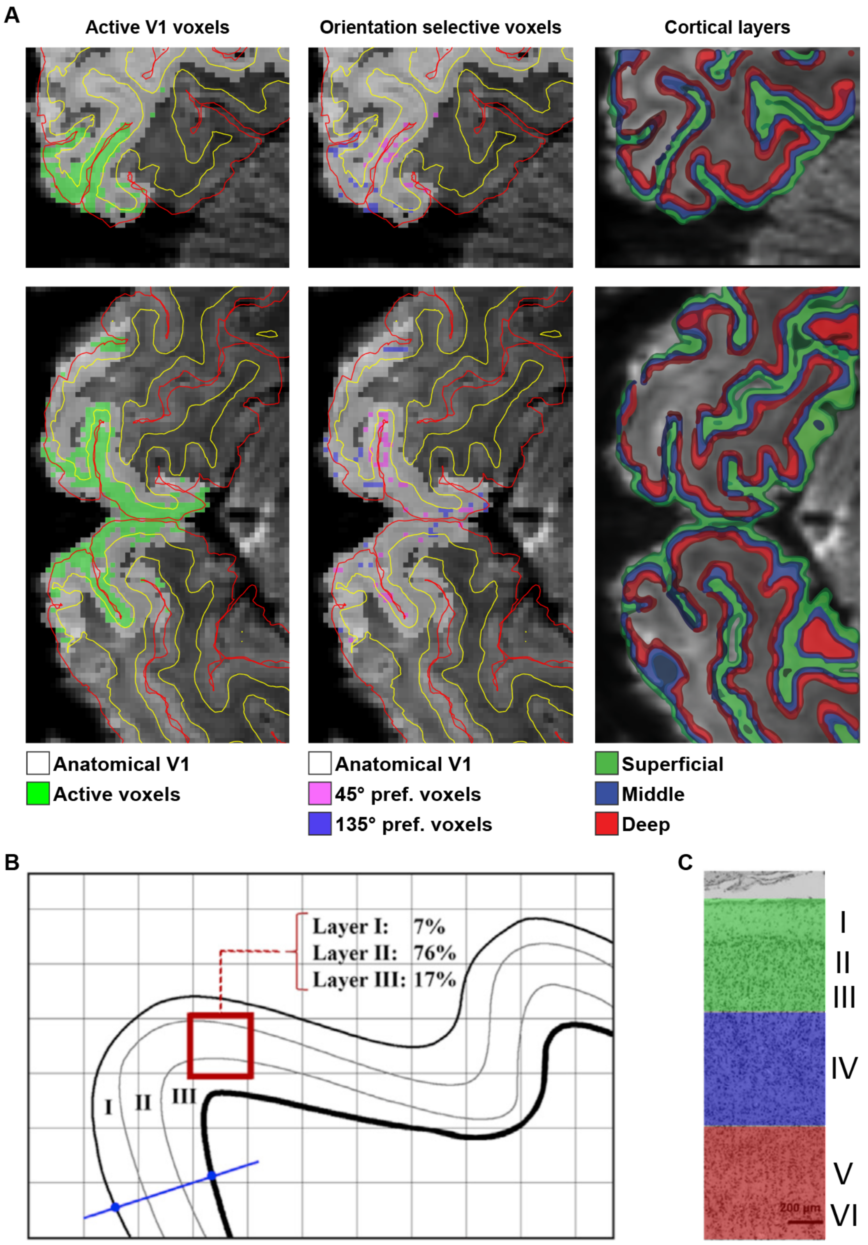

Figure 2. Analysis approach. (A) Illustration of ROI selection on sagittal slice of the mean functional scan of one participant. Within V1 (white), active voxels were selected based on significant activation in the functional grating localiser (green). From these active voxels, we selected the 500 most strongly 45°-preferring (pink), and 135°-preferring (blue) voxels, respectively. With all voxels in these two ROIs, we determined how their volume was distributed over the superficial, middle, and deep cortical layers. **(B)** Schematic example of a voxel (red square) and the distribution of its volume over the three gray matter layers. This layer-volume distribution was determined for each voxel and used as the basis of a regression approach in order to obtain layer-specific BOLD time courses (see Materials and Methods). **(C)** Deep, middle and superficial cortical layers indicated in coloured ribbons. Cytoarchitectural image of V1 adapted from (de Sousa et al., 2010).

Also, we have now quantified the laminar distribution of the selected voxels. Specifically, we determined for all voxels what proportion of their volume overlapped with the deep (16% +/- 1%, mean +/- SD over participants), middle (21% +/- 2%), or superficial (28 +/- 3%) layers. The fact that the superficial layer was more strongly represented in the set of selected voxels is in line with the bias towards superficial layers in GE EPI due to draining veins.

In order to investigate whether this overrepresentation of the superficial layers influenced our results, we repeated our analyses after correcting for this imbalance. That is, for each ROI, we retained a subset of the selected voxels such that all layers were represented equally, by iteratively removing voxels from the most overrepresented layer until a one-way ANOVA on the remaining voxels in a given ROI (with independent variable 'layer') no longer revealed a significant ($p > 0.1$) imbalance. This resulted in the exclusion of 80 (+/- 31, mean +/- SD) voxels per ROI on average. This control analysis yielded qualitatively the same effects as our main analysis (see Figure R2 below). That is, the laminar profile of orientation-specific BOLD signal evoked by expectations, in the absence of bottom-up input, was significantly different from that evoked by actually presented gratings ($F_{2,34} = 4.62$, $p = 0.017$). Specifically, expectations evoked orientation-specific BOLD signal in the deep ($t_{17} = 3.32$, $p = 0.0040$), but not in the middle ($t_{17} = 1.29$, $p = 0.21$) and superficial ($t_{17} = 0.10$, $p = 0.92$) layers. As expected, actually presented gratings evoked activity in all layers of V1 (deep: $t_{17} = 4.39$, $p = 0.00040$; middle: $t_{17} = 3.69$, $p = 0.0018$; superficial: $t_{17} = 3.41$, $p = 0.0034$). We now report the results of this control analysis in the manuscript (pp. 7-8).

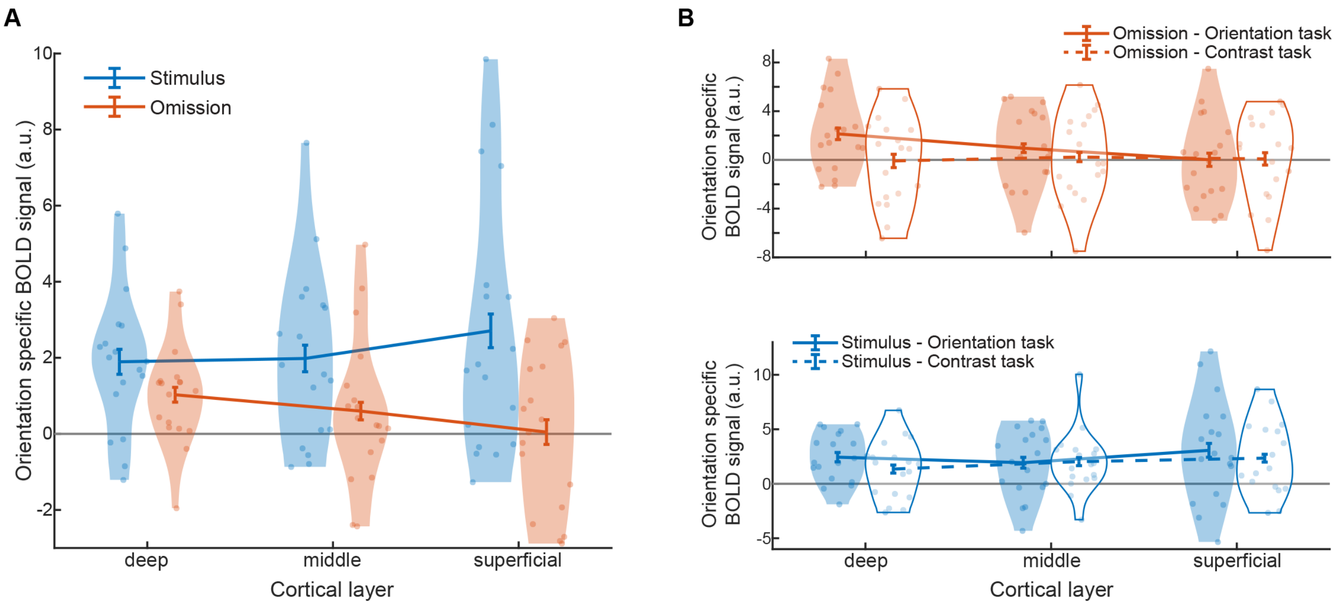

Figure R2. Orientation-specific BOLD response per layer, after balancing the laminar design matrices.

5. What are the consequences of the double-subtraction used to compute "orientation-selective" profiles? The analysis seems a bit circular: in voxels that responded more strongly to one orientation than another, there are stronger responses to that orientation. To convince readers that the orientation selectivity shown in Fig. 3 is not an uninteresting consequence of picking voxels that prefer one orientation and then subtracting out responses to the other orientation, additional information needs to be shown - as requested above, what are the spatial distributions of the 2 voxel sub-populations; in addition, what are the laminar profiles of the sub-populations' responses to the different stimuli, before subtraction?

First, it is important to point out that the voxel selection, i.e. selecting 45 degree preferring and 135 degree preferring V1 voxels, was done on the basis of independent data, namely from the flickering grating localiser run. Therefore, there is no circularity or ‘double dipping’ in this analysis.

Figure R3 below shows the response to both presented and expected-but-omitted stimuli before subtraction. As can be seen, BOLD responses are higher in subpopulations preferring the (expected) orientation in all layers for presented stimuli, and deep layers only for expected-but-omitted stimuli.

Note that the omission responses are overall negative before the subtraction. This is likely the result of the fact that we used a fast event-related design without an explicit baseline period. Specifically, in this type of design the baseline is effectively the mean signal, and when a stimulus is omitted, during a run in which stimuli are presented most of the time, the signal in V1 is likely to be lower than average. Essentially, this type of design is optimal for detecting differences between conditions (stimulus vs. omission, or 45 degree stimulus/omission vs. 135 degree stimulus/omission), which was our main interest here, but suboptimal for detecting main effects of single conditions (e.g. stimulus vs. baseline, or omission vs. baseline). With this lack of interpretability in mind, we suggest not to include the figure below in the manuscript, but we are happy to do so should the reviewer prefer this.

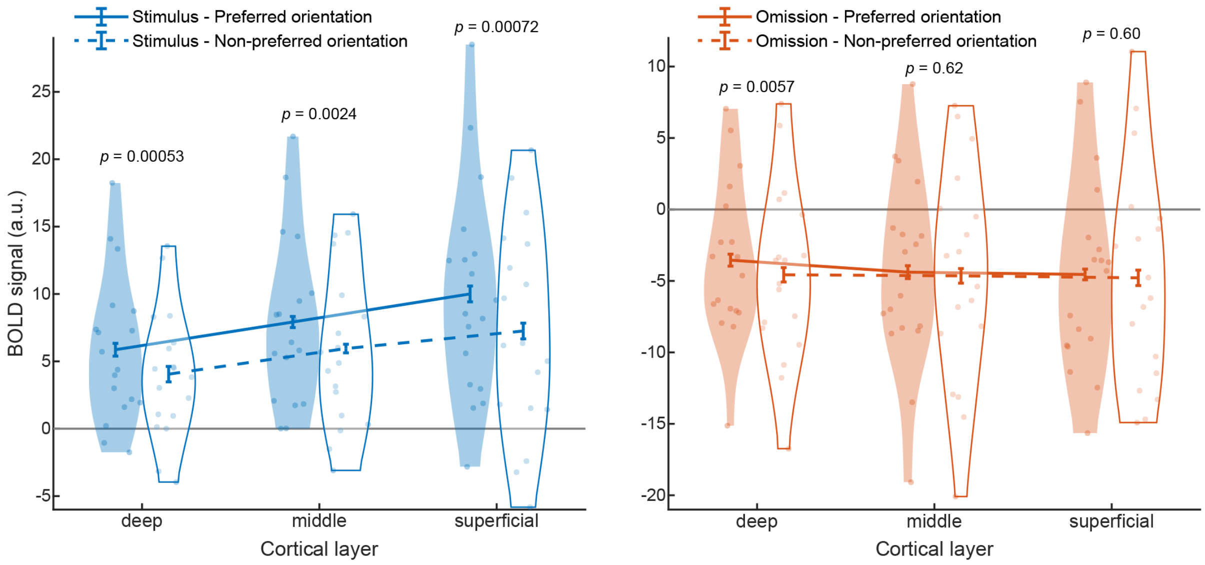

Figure R3. Stimulus and omission responses in V1, separately for voxel sub-populations preferring (solid lines, filled shapes) and non-preferring (dashed lines, open shapes) the current orientation. Error bars indicate within-participants SEM.

6. The Results section does not mention the results of the various ANOVAs that are described in the Methods.

We have now improved the description of the statistical in the Methods, and have also expanded our reporting of the results of these tests in the Results (pp. 6-7). (See the response to Reviewer 4 for specific details.)

7. The Results section describes the analysis of V1 sub-populations, but does not include a statement that these were then averaged to generate a laminar profile. That statement is present in the Methods (line 335), but it will save readers a moment of confusion if you can include a similar statement in the Results.

We thank the reviewer for pointing this out, and we have now included such a statement in the Results (p. 6). We agree that this makes the Results section clearer.

8. Which results are shown in Fig. 2? Are these collapsed across all 4 experimental runs, so "expected" and "seen" profiles reflect average results from the orientation task and the contrast task? It would be very helpful if you would put a clarifying statement in the figure legend. It seems likely that the orange trace in Fig. 2 is the combination of the 2 orange traces in Fig. 3, but it would be nice to know for sure.

Figure 2 in the previous version of our manuscript – now Figure 3A – indeed showed the results collapsed over all 4 experimental runs. We have now made this explicit in the figure legend. Also, the new Figure 3B shows the results split out for the two tasks. The top panel of Figure 3B replaces the old Figure 3, and the bottom panel is new. We feel that this new figure, showing the results averaged over tasks (Figure 3A) side by side with the results split by task (Figure 3B), gives a clearer overview of the results.

*9. The predictive coding theories that I'm familiar with suggest that prediction signals that are present in deep layers would be fed back to both deep and superficial layers of upstream areas. So if V1 is representing a *received* prediction on "unseen" trials, not all theories suggest that it would be present only in deep layers. On the other hand, if V1 is *generating* a prediction on expectation-only trials, that would explain the pattern that is measured. But if that's the case, then either feedback to sub-cortical targets would be the only place that prediction would go or V1 is breaking the dogma of feedforward connections arising in superficial layers and traveling to downstream middle layers. Felleman and Van Essen leave room for that possibility, but it would be nice to see some deeper discussion in the present manuscript about exactly why prediction signals are present in deep layers of primary visual cortex.*

We thank the reviewer for raising this interesting point. We do indeed interpret our findings as top-down signals received, rather than generated by V1, since the predictions were induced by arbitrary associations between coloured cues and grating orientations that were learnt only minutes before the participants were scanned. Cortico-cortical feedback connections preferentially target layers 1 and 5/6 in upstream regions (Rockland and Pandya, 1979; Harris and Mrsic-Flogel, 2013). Feedback connections to L5/6 are of course an excellent candidate mechanism for the prediction signals reported here. Feedback signals in L1 are less likely to be directly detectable in fMRI, since L1 is very thin and sparsely populated with neurons. Rather, feedback to L1 is likely to modulate activity in other layers. That is, feedback to L1 targets dendritic tufts of pyramidal cells in L2/3 and L5 extending into L1 (Harris and Mrsic-Flogel, 2013). These feedback connections, targeting apical dendritic tufts far from the soma, are unlikely to drive activity in pyramidal neurons by themselves, but have rather been suggested to have a modulatory function, e.g. coincidence detection (Larkum, 2013). However, in the omission trials in the current study there are no sensory inputs to be modulated. This might explain why in the current study we observe (driving) effects of feedback to deep layers, but no (modulatory) effects in the superficial layers of V1. This is in line with the findings in Kok et al. 2016, where feedback in the absence of bottom-up input activated only the deep layers of V1 (as here), but feedback in the *presence* of bottom-up input led to modulations in both deep and superficial layers (Kok et al., 2016). Therefore, we predict that if instead of omitting the predicted grating stimuli we would have presented gratings with unexpected orientations, we would observe modulations in both deep and superficial layers. Future studies will be needed to test this hypothesis.

In the context of predictive coding, modulatory feedback connections to L1 serve the function of calculating prediction errors, i.e. modulating activity in L2/3 pyramidal neurons that receive (non-)predictable sensory inputs (Bastos et al., 2012). Driving feedback connections to deep layers may serve to excite predictions in upstream regions consistent with the downstream ones (Spratling, 2008). Note that considerations of the modulatory and driving roles of feedback connections are not exclusive to predictive coding theories, but instead are pertinent to any theory on the role of feedback in perception. Indeed, we feel it’s important to point out that our findings are not exclusive support for predictive coding theories, but are in line with other theories that suggest that predictions play a crucial role in sensory processing as well (e.g., Lee and Mumford, 2003). In future work, it will be crucial to test whether or not cortex indeed calculates explicit prediction errors during sensory processing, as proposed by predictive coding theories.

We have now expanded our Discussion to include these points.

Reviewer 2

I found the manuscript to be nicely written and clearly laid out, but the level of detail in the data presentation just too insufficient to support the claim that anything related to stimulus expectations is being measured.

There are basically just two times 6 data points (+/- SE) presented in this paper?

The methods state that there were 18 participants in the study, for each of which a serious amount of data analysis was done to get to this summary (cortical segmentations, region of interest definitions [early visual cortical areas], division into "orientation-selective" groups of voxels, ...). The authors should present at least a bare minimum of data summaries for those - not just for the review process, but also for readers of the article to be able to judge the reliability of individual steps in the analysis.

I find this area of research fascinating and I'd be very interested in seeing a more detailed revision of this manuscript, but this current version of presenting the result is just too condensed...

We apologise for not providing enough information in the original manuscript. We presented our findings in a Short Report, since we felt the main finding lent itself to it, and our methodology closely matched that used by some other recent studies (Lawrence et al., 2018, 2019). However, we now recognise that we should have provided a lot more detail, and have greatly expanded our manuscript.

- We have added a new Figure 2 visualising the analysis, as well as ROIs from a representative subject.
- We have added a new Supplemental Figure 1 visualising the registration between anatomical boundaries and functional images for all participants.
- A new Supplemental Figure 2 visualises the temporal GLM approach, and how it is used to calculate layer-specific, orientation-specific BOLD signals.
- The new main results figure (Figure 3) now shows individual datapoints, and their distribution (density) using violin plots.
- Finally, we have included two supplementary figures (S3 and S4) showing the results of two control analyses, instilling additional confidence in our results.

Additionally, we have expanded our description of the analysis in the Materials and Methods, and our Discussion of the results. We hope the reviewer now finds this strongly elaborated manuscript appropriate for review.

Reviewer 3

1. Leaving the aside the robustness issue for the moment and taking the data at face value, the authors' conclusion that the signals in the deep layers reflect "stimulus templates" - whatever that means - seems rather arbitrary. They could equally be described as evidence of feature-based and/or spatial attention, a novelty or surprise signal, or some combination thereof. Equally, one could use the term "imagery" to refer to these signals. Curiously, the authors previously reported activity in deep layers by illusory figures but ascribe those signals to "automatic structural expectation" when they would readily be explained by attentional mechanisms as well. Overall, the discussion is rife with speculative reasoning (e.g. the comparison with working memory) and conflate expectation signals (i.e. the actual BOLD signal measured to an omitted stimulus) with evidence of "expectation templates" (which is an abstract concept that is never defined and is just the authors interpretation of those signals). The term only makes sense within the framework of predictive coding theories, which remain largely speculations with little or no solid experimental support from actual neuronal recordings. [The issues with predictive coding theories are somewhat beyond the scope of this paper, but I would like to see a proponent of these theories explain why a mechanism that purports to be fundamental to perceptual processing and uses 90% of all corticocortical connections, seems only to be active in human cortex (even though macaques have just as many feedback connections), can only be measured with fMRI, and is primarily evident for highly learned shapes...] The authors need to strongly tone down the speculation here and refrain from using nebulous terms like "stimulus templates" in the results and titles - if they wish to speculate about this in the discussion then the term needs to be defined and alternative interpretations considered. Since I don't see how these alternative interpretations can be ruled out, I don't think their conclusions are robustly or uniquely supported by the data.

We thank the reviewer for raising these points.

We agree that we did not clearly define what we meant by ‘stimulus templates’. What we were referring to with this term is the fact that merely expecting a stimulus evoked a pattern of activity in sensory cortex that resembles the pattern of activity evoked when that stimulus was actually presented. One potential way for this to occur is by top-down modulations increasing the firing rate of neurons tuned for the expected stimulus feature (e.g. 45° orientation). However, we realise that this term is open to interpretation and speculation, and have therefore changed it to ‘stimulus-specific activity’ in the title and throughout the manuscript.

We also agree that it is important to consider whether the mechanisms underlying our expectation manipulation are distinct from or identical with those underlying other top-down phenomena, including feature-based attention, working memory, and imagery. Specifically, mechanistically, the stimulus-specific signals we observe in V1 may be the result of top-down gain on neurons representing the orientation, similar to the mechanism suggested to underlie working memory (Harrison and Tong, 2009; Serences et al., 2009), imagery (Albers et al., 2013) and feature-based attention (Treue and Trujillo, 1999; Serences and Boynton, 2007; Jehee et al., 2011). In fact, this is why we directly compared our results with those of two recent studies using virtually identical methodology (as we now point out in the Discussion) to investigate layer-specific orientation-specific BOLD signals induced by working memory (Lawrence et al., 2018) and feature-based attention (Lawrence et al., 2019). It is interesting to note that these top-down processes had a different laminar profile from the effects we report here (specifically, the involvement of the superficial layers in those studies), suggesting that the underlying neural mechanisms may differ. Of course this is speculative at this stage, and we now make sure to emphasise this in the Discussion, but we feel it is a worthwhile comparison to make.

Similarly, the comparison with the illusory figure effect with a similar laminar profile in a previous study (Kok et al., 2016), while speculative, we feel is interesting. Note that those previous illusory figure effects are not straightforwardly attributable to attention, since attention was manipulated in that study and the illusory figure effects were found to be independent of spatial attention. As for the point above, we now explicitly acknowledge that this comparison is speculative.

Regarding predictive coding, we first wish to point out that we do not claim that our results uniquely support predictive coding theories. Our results are certainly in line with these theories, but they are equally in line with other theories that propose an important role for predictive feedback in perceptual inference (e.g., Lee and Mumford, 2003; Raizada and Grossberg, 2003). Second, while we certainly agree that direct evidence for predictive coding is currently lacking (as we now make explicit in our manuscript), there is a substantial body of evidence for effects of expectations induced by statistical regularities on neural firing in macaques (Meyer and Olson, 2011; Meyer et al., 2014; Bell et al., 2016; Kumar et al., 2017; Schwiedrzik and Freiwald, 2017; Kaposvari et al., 2018) and rodents (Fiser et al., 2016; Zmarz and Keller, 2016; Vinken et al., 2017). Some studies have even reported coding of prediction- and prediction error-like responses in distinct populations of neurons (Bell et al., 2016; Fiser et al., 2016; Jordan and Keller, 2020), though this issue is certainly far from settled. In sum, we respectfully disagree with the reviewer that evidence for effects of predictive processing are only found in human fMRI, but certainly agree that our findings are not uniquely attributable to predictive coding, which we now make explicit in our Discussion. We also now emphasise in our Introduction that evidence for modulations of sensory processing by prior expectations comes from both non-invasive human neuroimaging as well as animal electrophysiology, which we believe has improved the paper.

2. With regard to robustness, laminar fMRI remains a rather speculative endeavour and many questions remain about its reliability. Given what is known about cortical blood supply and regulation, there are many reasons to be sceptical about strong claims of interlaminar differences in BOLD response (especially using gradient echo rather than spin echo). It is also clear that there is a great deal of heterogeneity in the literature about laminar differences, even for studies that are quite similar (e.g. cf the Lawrence at al 2019 study with this and the Kok et al 2016 paper). Laminar differences could conceivably be due to a whole slew of mechanisms, such as differences in blood supply and regulation, neurovascular coupling, contribution of draining venules and vein etc. This uncertainty is compounded by the lack of hard electrophysiological reference data for this type of experimental paradigm (specifically, there is little or no compelling evidence for expectation effects in macaque cortex, certainly not in V1, and less still for interlaminar differences in firing rates). It would have been more convincing had the authors demonstrated clear laminar differences in their setup for a stimulus known to evoke layer-specific differences in response (e.g. monocular vs binocular stimulation).

We certainly agree that laminar fMRI is a novel method that will require more validation and development. Exciting developments are underway to try to account for known biases in cortical blood supply and regulation, such as the fact that blood is drained towards the cortical surface (Heinzle et al., 2016; Markuerkiaga et al., 2016, 2020). Nevertheless, it is encouraging to see that many laminar fMRI studies report effects in line with known physiology as well as animal studies, where available. For instance, Lawrence and colleagues (2018) reported effects of working memory in deep and superficial layers of V1, in line with macaque studies (Van Kerkoerle et al., 2017), while stimulus contrast most strongly modulates responses in the middle layers (Lawrence et al., 2019), as would be expected given that L4 is the input layer. As another example, Fracasso and colleagues (2016) report that estimated V1 centre and surround receptive field sizes are smallest in the middle layers and larger in agranular layers, in line with reports of macaque V1 (Bijanzadeh et al., 2018).

Finally, in a recent preprint, Hollander et al. (2020) report strongest ocular dominance effects in the middle layers, in line with the seminal work by Hubel & Wiesel in macaque V1 (Hubel and Wiesel, 1968). We now point out these correspondences in our Discussion, while also acknowledging the need for studies using paradigms similar to the one used in the current study and other human laminar fMRI studies to corroborate these results. To our minds, human neuroimaging and animal neurophysiology are strongly complementary, and layer-specific fMRI and other high-resolution neuroimaging methods provide an exciting opportunity to start bridging the gap between the two approaches.

We do wish to point out that, as in most previous layer-specific fMRI studies, we directly compare two different experimental conditions (stimulus present vs. stimulus expected-but-omitted) in the exact same voxels, ruling out differences in physiology (e.g. structure and density of veins) as a potential explanation for the different laminar profiles in the two conditions. We now make this explicit in our Discussion, and emphasise the need for such control or baseline conditions in any laminar fMRI study, given the problems with interpreting a single laminar BOLD profile on its own (p. 12):

> "Layer-specific fMRI is a novel technique that is not without its challenges, such as the non-linear relationship between layer-specific neural activity and the BOLD signal, due to the complex microvasculature of cortex (e.g., draining veins). For this reason, it is crucial to compare laminar profiles between experimental conditions that isolate a specific effect of interest, since a single laminar BOLD profile can be challenging to interpret. Encouragingly, results from studies that have done so have generally been in good alignment with invasive animal electrophysiology (Fracasso et al., 2016; Huber et al., 2017; Self et al., 2019). For instance, Lawrence and colleagues (Lawrence et al., 2018) reported effects of working memory in deep and superficial layers of V1, in line with macaque studies (Van Kerkoerle et al., 2017), while stimulus contrast most strongly modulated responses in the middle layers (Lawrence et al., 2019), as would be expected given that layer 4 is the main bottom-up input layer. Finally, Fracasso and colleagues (Fracasso et al., 2016) reported that estimated V1 centre and surround receptive field sizes are smallest in the middle layers and larger in agranular layers, in line with reports of macaque V1 (Bijanzadeh et al., 2018). Additionally, efforts are ongoing to correct for known effects of vasculature using sophisticated analysis techniques (Heinzle et al., 2016; Markuerkiaga et al., 2016). Further research will be needed to corroborate the results from human layer-specific fMRI studies using invasive electrophysiology. Human neuroimaging and invasive neurophysiology are highly complementary approaches that often do not sufficiently interact, and layer-specific fMRI and other high-resolution neuroimaging methods provide an excellent opportunity to start bridging this gap."

We acknowledge the heterogeneity in results between laminar fMRI studies, which in some cases may lead to confusion, especially when acquisition and analysis approaches differ strongly between studies. However, when these are closely matched, such as between the current study and the references studies on working memory (Lawrence et al., 2018) and feature-based attention (Lawrence et al., 2019), differences in layer-specific activity can actually be informative, as we discuss in reply to point 1 above. While it's currently far too early to be confident in the exact differences in neural mechanisms between expectation, working memory, and feature-based attention that may underlie the differences between these studies, making the comparison and engaging in some theorising can lead to novel hypothesis to be tested in future research.

Finally, we now emphasise the need for electrophysiological laminar work to follow up on our findings. Until recently, not many researchers have investigated these type of questions (i.e. the role

of expectation/prediction in perception) in macaques and other animal models, but this is now starting to change, most notably with the excellent work done by the Georg Keller (Fiser et al., 2016; Zmarz and Keller, 2016; Keller and Mrsic-Flogel, 2018; Jordan and Keller, 2020) and Andre Bastos (Bastos et al., 2015, 2020) (but also others, see reply to point 1 for further examples). Specifically, while still a pre-print at this stage, a recent study by Andre Bastos in macaques found that "Pre-stimulus predictions were associated with increased alpha/beta (8-30 Hz) power/coherence that fed back the cortical hierarchy primarily via deep-layer cortex" (Bastos et al., 2020), which is very much in line with our findings here.

3. It is possible that my concerns are overstated, but it does not lend a great deal of confidence in the results that the presented data are so highly processed - they are the result of serial estimations that hinge critically on a) near-perfect alignment of anatomical and structural data (which seems doubtful - and the authors present no evidence of the accuracy of the alignment) b) the accuracy and reliability of their method to extract laminar-weighted responses, and c) that the GLM fits the evoked responses faithfully (again, we are not shown any raw data or goodness-of-fit metrics). The results would be a lot more compelling if they could be shown without such excessive data cleaning. In short, extraordinary claims require extraordinary data.

We appreciate the reviewer's concerns and now provide additional data and visualisations to address these.

First, the new Figure S1 shows the alignment of anatomical boundaries to functional images for all individual participants, both before and after the recursive boundary registration (RBR) (Van Mourik et al., 2019) we performed to compensate for local distortions in the EPI images. This recursive boundary registration, performed subsequent to a 'standard' whole brain rigid-body BBR, increased the average contrast across the gray-white matter boundary from -0.0060 (+/- 0.0022, mean +/- SD) to -0.0100 (+/- 0.0024, mean +/- SD), an increase of 75%. (Note that the contrast values are negative since white matter is darker than gray matter on T2* images.)

We also validated the placement of these boundaries, as well as the laminar extraction method, by quantifying the raw functional image intensity for the three gray matter layers, as well as white matter and CSF. Note that these results were obtained from the exact same data as our main results, by simply averaging the EPI time courses extracted from our ROIs (V1 45 degree preferring voxels and V1 135 preferring voxels, respectively, averaged together for this purpose) by the layer-specific spatial GLM. As expected for T2* weighted images, the signal intensity was higher in the three GM layers (deep: 455 +/- 80; middle: 456 +/- 86; superficial: 461 +/- 80; mean +/- SD over participants) than in WM (427 +/- 83; lower than mean GM: t_{17} = 5.17, p = 7.7 x 10^{-5}) and outside of the brain (430 +/- 78; lower than mean GM: t_{17} = 3.81, p = 0.0014). See Figure R1, in the response to Reviewer 1, point 3, for a visualisation. We now report this quantification in the manuscript.

Finally, addressing point c) raised by the reviewer, we now show an example of the fit of the temporal GLM in the new Figure S2 (reproduced below for convenience), and report goodness-of-fit per cortical layer (deep: R^2 = 0.43 +/- 0.09, middle: R^2 = 0.44 +/- 0.08, superficial: R^2 = 0.46 +/- 0.09; mean +/- SD over participants).

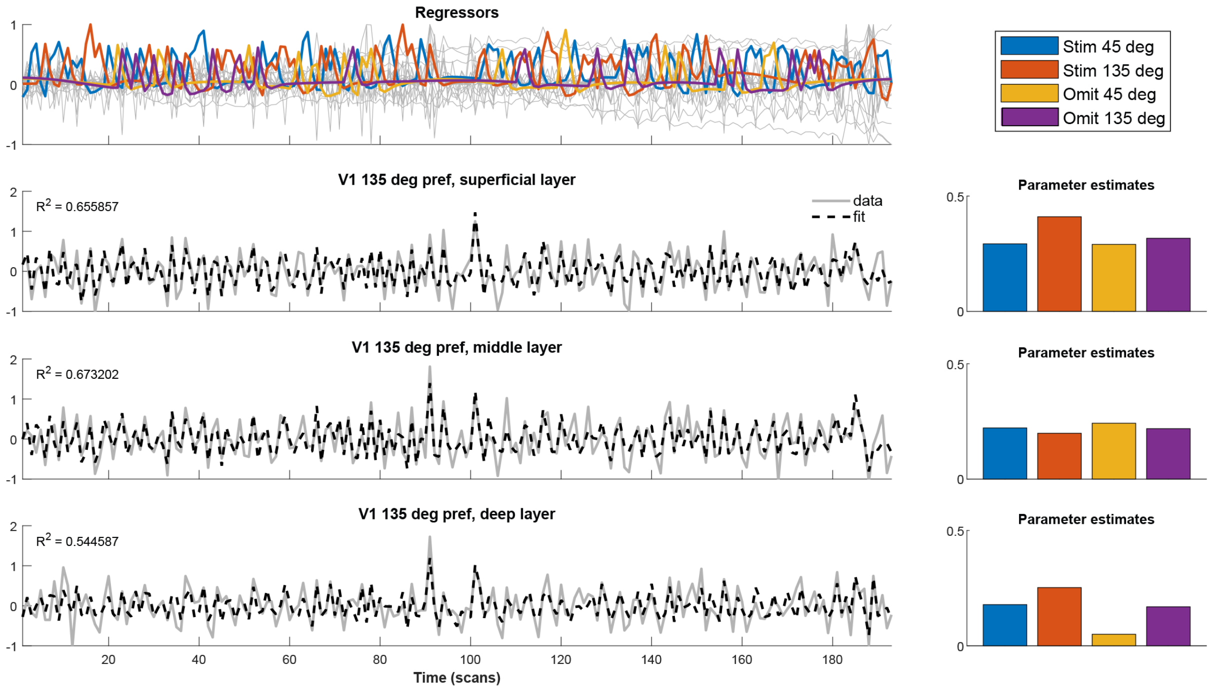

Figure S2. Illustration of temporal GLM. Example model and data shown for one participant (P1) and one ROI (V1, 135 degree preferring voxels). Left, top panel: regressors used in the temporal GLM. Coloured time courses indicate regressors for the four conditions of interest, gray time courses indicate nuisance regressors (i.e., head motion). Left, three bottom panels: fMRI time courses in each of the three gray matter layers (solid gray) and time courses fit by GLM (dashed black). Right, three bottom panels: parameter estimates for the four regressors of interest, quantifying the amplitude of the BOLD response evoked by the four conditions. There parameter estimates constitute the main results as shown in, e.g., Figure 3.

4. The authors have not defined V1 by retinotopy, and anatomy is only an approximate predictor of cortical topography. The authors should refrain from referring to their ROI as V1 - more honest would be to say calcarine cortex, or "anatomically defined V1".

Indeed, we defined V1 by anatomy, which has been shown to be a reliable method of identifying V1 (Hinds et al., 2008). We now use the suggested terminology in our manuscript (p. 5):

> "We non-invasively examined the laminar profile of the activity evoked by these orientation expectations in anatomically defined human V1, …"

5. Figures 2 and three state that "Error bars indicate within-subject SEM". It is not clear what this means - is it the average of within-subject SEM (based on what average?) Or is it across-subjects?

The within-subject SEM quantifies the across-subject variability of the differences between conditions, rather than the across-subject variability of the individual conditions (Cousineau, 2005; Morey, 2005). That is, the across-subject variance of the overall mean across conditions, which is not relevant in a within-subjects design, is removed when calculating the SEM. This is the appropriate approach to visualising across-subject variance for within-subject designs such as the one used in the current study, since what we are interested in is how reliable the differences between conditions are (i.e., between layers and between stimulus vs. omission), rather than the variability of the mean BOLD response across conditions. We now realise we did not make this clear in our original manuscript, and have added this information and the relevant references to the Materials and Methods section.

6. The methods are rather opaque, but it would seem that what is reported are not actual BOLD signals but beta estimates from the GLM. This needs to be stated clearly, and the authros need to show that the results based on raw BOLD measurements agree with those basec on the beta estimates (which hide the underlying variability).

We apologise for not description the methodology sufficiently clearly. We have now expanded this description in many places, and have added new figures to visualise our approach (Figure 2 and Figure S2). Figure S2 particularly visualises how the results displayed in the main results are estimated; through a temporal GLM on layer-specific time courses. We now also make this explicit in our manuscript (p. 21):

> "The parameter estimates for the regressors of interest were the basis of our main analyses, described below."

Note that we cannot perform our main analyses fully on raw BOLD instead of parameter estimates, given the temporally overlapping BOLD responses to individual trials in a fast event-related design. In a fast event-related design (i.e. ITI < ~15-20s) a temporal GLM is required to disentangle the BOLD responses to the different conditions, as illustrated in Figure S2. Note that is not unique to our study, but applies to any fMRI study that does not separate trials by ~15-20 seconds. However, we now perform analyses that stay closer to the raw data by repeating the analyses without weighting voxels by t statistics (reply to point 7, below) and without performing the layer-specific GLM (reply to point 8, below).

7. I would like to see how robust the results are withouth weighting the results by t statistics

We have now performed this analysis, and included the results in the manuscript and a new Figure S3 (reproduced below). Importantly, this analysis reproduced our main results (cf. Figures 3 and S3) (pp. 8-9):

> "For our main analysis, individual voxel time courses were normalised and weighted by how orientation-selective they were (see Materials and Methods for details), before applying the layer-extraction. In a control analysis, we conducted our analyses on the raw voxel time courses, omitting the normalisation and weighting steps. This analysis qualitatively reproduced our main results: the laminar profile of orientation-specific BOLD signal evoked by expected-but-omitted gratings was significantly different from that evoked by actually presented gratings ($F_{2,34}$ = 8.68, p = 0.00090; Figure S3). As in our main analysis, expectations evoked orientation-specific BOLD signal in the deep (t_{17} = 3.30, p = 0.0042), but not in the middle (t_{17} = 0.21, p = 0.84) and superficial (t_{17} = 0.22, p = 0.82) layers, while actually presented gratings evoked activity in all layers of V1 (deep: t_{17} = 4.13, p = 0.00070; middle: t_{17} = 2.88, p = 0.010; superficial: t_{17} = 3.71, p = 0.0017)."

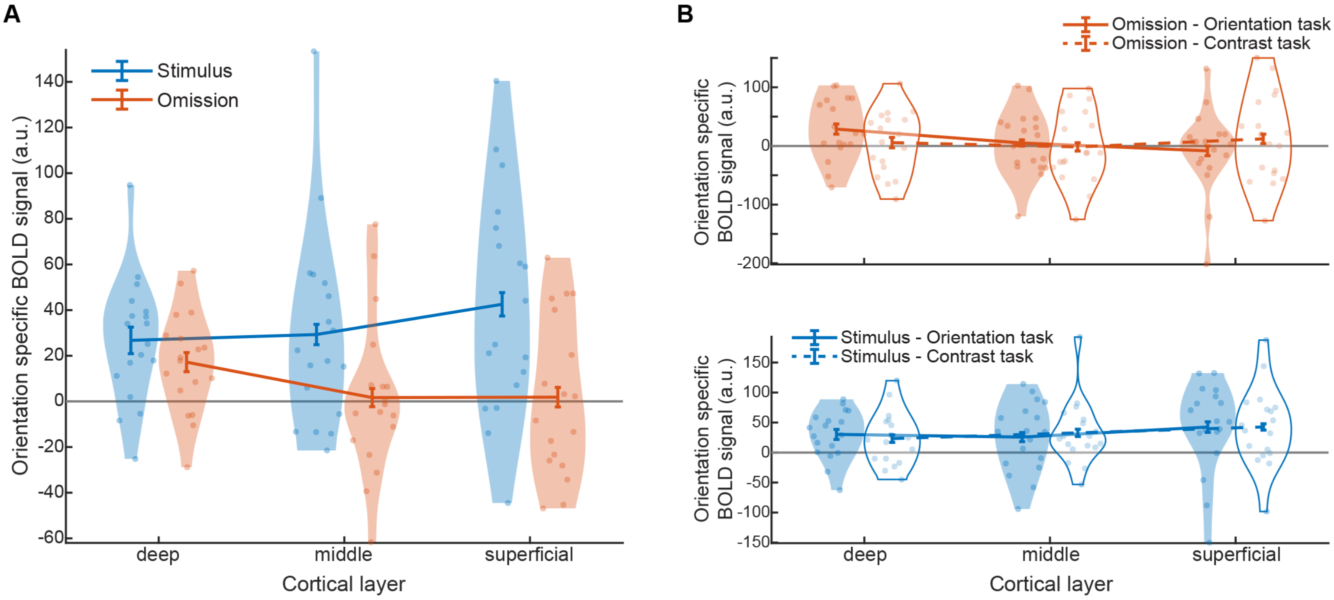

Figure S3. Layer-specific BOLD response in V1 for presented and expected stimuli, based on raw voxel time courses. Normalising and weighting voxel time courses by orientation selectivity was omitted in this control analysis. **(A)** Orientation-specific BOLD response to presented (blue) and expected-but-omitted (orange) gratings in the different layers of V1, averaged over tasks. **(B)** Orientation-specific BOLD response to expected-but-omitted (orange, top panel) and presented (blue, bottom panel) gratings, separately for the orientation (solid lines, filled shapes) and contrast (dashed lines, open shapes) tasks. Dots represent individual participants, curved shapes indicate density. Error bars indicate within-subject SEM.

8. The extraction of laminar-specific time courses relies on a lot of unspecified parameters (e.g. the correlation length parameter) and we are not told how well this parameterisation fits the data. Moreover this method has been shown to be sensitive to noise (Mourik et al) compared to a simple interpolation. The authors need not only to report the full methods for determining the laminar design matrix and the values used, but show that their results are not dependent on the use of this method.

We have now greatly expanded our description of the definition of the cortical layers and the extraction of layer-specific time courses in the Materials and Methods (pp. 19-21). In short, the laminar $n \times k$ design matrix **X** represents the layer volume distribution, i.e. the distribution of the k layers over the n voxels within the ROI. Every row of **X** gives the distribution of a given voxel volume over the layers and every column (regressor) represents the volume of the corresponding layer across voxels. The ordinary least squares (OLS) regression of the voxel signals against the design matrix yielded the layer signals. We previously did not specify that the regression method used for the spatial GLM was OLS, which does not rely on parameters like correlation length; this has now been amended.

To rule out that our results were driven by the specifics of the laminar regression method, we have now repeated our main analyses on layer-specific time courses extracted through interpolation rather than a spatial GLM:

> "In a control analysis, we extracted laminar time courses using an interpolation method rather than a spatial GLM. Interpolating a volume at different cortical depths effectively weights for all voxels in an ROI with respect to the layers:

$$\widehat{\mathbf{B}}_{interpolation} = \mathbf{X}^{\mathbf{T}} \cdot \mathbf{Y}/N$$

N is the number of voxels. The results of this analysis qualitatively replicated those of the main analysis, and are presented in Figure S4."

We describe the results of this control analysis in the Results section (p. 9) and in a new Figure S4 (reproduced below):

"To rule out that our results were driven by the specifics of the laminar regression method, we repeated our main analyses on layer-specific time courses extracted through interpolation rather than a spatial GLM (see Materials and Methods for details). As for the other control analyses, this yielded qualitatively the same results as our main analysis: the laminar profile of orientation-specific BOLD signal evoked by expected-but-omitted gratings was significantly different from that evoked by actually presented gratings ($F_{2,34}$ = 9.11, p = 0.00068; Figure S4). This was driven by the fact that expected-but-omitted gratings evoked orientation-specific BOLD signal in the deep (t_{17} = 2.74, p = 0.014), but not in the middle (t_{17} = 1.17, p = 0.26) and superficial (t_{17} = 1.00, p = 0.33) layers, while actually presented gratings evoked activity in all layers of V1 (deep: t_{17} = 4.23, p = 0.00057; middle: t_{17} = 4.13, p = 0.00070; superficial: t_{17} = 4.27, p = 0.00052)."

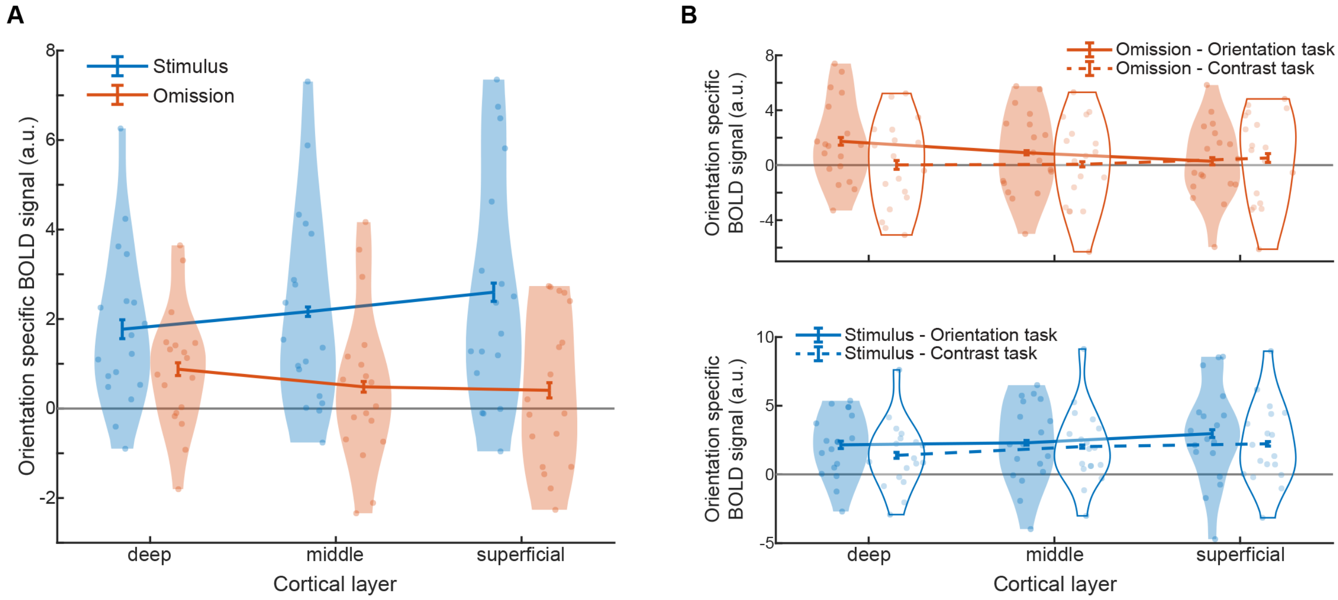

Figure S4. Layer-specific BOLD response in V1 for presented and expected stimuli, determined by interpolation rather than spatial GLM. (A) Orientation-specific BOLD response to presented (blue) and expected-but-omitted (orange) gratings in the different layers of V1, averaged over tasks. **(B)** Orientation-specific BOLD response to expected-but-omitted (orange, top panel) and presented (blue, bottom panel) gratings, separately for the orientation (solid lines, filled shapes) and contrast (dashed lines, open shapes) tasks. Dots represent individual participants, curved shapes indicate density. Error bars indicate within-subject SEM.

Note that we also performed such a control analysis in Kok et al. 2016, where the interpolation method also yielded similar results as the laminar regression method. The main difference between the two methods seems to lie in the fact that the correlation between the time courses of the different layers as determined by the interpolation method is higher as compared to the spatial GLM method, suggesting increased unmixing of the layer signals by the GLM approach.

9. Much more details are needed about the variability in segmentation and ROI definitions across subjects. Specifically the authors need to show that the results are not driven by a small number of

subjects and that the pattern seen across individuals is replicable within individuals. Rather than error bars the authors should show individual subject means.

We now provide much more data on the individual participants. First, as mentioned above, we now visualise the registration between anatomical boundaries and functional images for all individual participants (Figure S1). Second, all result figures now show the individual participant means (Figures 3, S3, and S4). We feel that this has greatly enriched the presentation of our results, and we thank the reviewer for this suggestion.

Reviewer 4

1. The authors compare stimulus vs. the omission conditions and report a significant interaction between conditions and layers. For the stimulus condition, Figure 2 shows higher BOLD in the superficial layers, which is consistent with the known superficial bias for GE BOLD. Could this superficial bias drive the significant interaction (condition x layer) reported as the key finding of this study? To demonstrate layer-specificity, it is important to test whether there are significant differences in BOLD across layers in the omission condition (i.e. one-way ANOVA showing higher BOLD for deep than middle and superficial layers).

We thank the reviewer for raising this point. We think that the interaction between 'layer' and 'stimulus vs. omission' is the crucial omnibus test to perform since a single laminar profile is hard to interpret, precisely because of vascular issues like the draining veins that cause the superficial bias in GE BOLD. However, we certainly agree that it is valuable to test laminar differences within the omission condition to follow up this interaction, and we have now done so using post hoc t-tests (p. 6):

> "In fact, the expectation-evoked orientation-specific BOLD signal was significantly stronger in the deep layers than in the middle (t_{17} = 2.8, p = 0.012) and superficial (t_{17} = 2.5, p = 0.025) layers."

We feel that these additional tests have strengthened our results.

2. The omission data in Figure 2 and omission-orientation task data in figure 3 should be the same data. However, the values don't match across figures; it looks as if the data presented in Figure 2 is the average data across tasks (orientation, contrast) presented in Figure 3. As the authors highlight that the two tasks showed a different pattern of results, these data should not be averaged. Does BOLD differ significantly across layers for the omission-orientation task?

We apologise for this confusion. The omission results in our previous Figure 2 were averaged over the two tasks, whereas the results in previous Figure 3 was split between the two tasks. In other words, the omission results in Figure 2 were the average over the results in Figure 3, as the reviewer suspected. To present our results more clearly, we have now organised both our figures and our statistical approach differently.

Rather than performing two 2-way repeated measures ANOVAs, one aimed at testing the interaction between stimulus type (presented vs. omitted) and layer, averaging over task, and one aimed at testing the effects of the task, we now test all three factors in a 3-way repeated measures ANOVA. This is of course the appropriate way of testing our results, and we thank the reviewer for encouraging us to scrutinise our statistical approach. Our main interest here, and the most robust interaction effect in our 3-way ANOVA, was the interaction between stimulus type (presented vs. omitted) and layer ($F_{2,34}$ = 5.4, p = 0.0093). This interaction was also replicated in all control analyses we performed (pp. 7-9). The 3-way interaction, between stimulus type, layer, and task (orientation vs. contrast task) was also significant ($F_{2,34}$ = 3.3, p = 0.048). However, given that this effect was less robust (i.e., it did not attain significance in all control analyses), and it was not our main effect of interest, we chose to focus first on the robust 2-way interaction between stimulus type and layer and then on the less robust 3-way interaction secondly. This order of priorities is also reflected in our new results Figure 3, in which the results are presented both averaged over tasks (in panel A) and separately for the two tasks (in panel B). By making this explicit in the Figure legend, and presenting the averaged and split results side-by-side, we hope the reviewer agrees the presentation of the results is now a lot clearer.

3. Could the difference between the stimulus and omission conditions be due to differences in the task? Participants performed the orientation task only for the stimulus but not the omission trials. Comparing the omission trials across tasks (orientation, contrast) is a fairer comparison, but it yields a weak effect showing no-significant differences between tasks in deep layers. It would be more convincing to test for BOLD differences across layers for the omission-orientation task.

We take the reviewer to suggest that the 3-way interaction between stimulus type, layer, and task might be driven by the stimulus present condition, but not the omission condition. This is an interesting suggestion. To look into this, we conducted 2-way repeated-measures ANOVAs with factors task (orientation vs. contrast task) and cortical layer (deep, middle and superficial), separately for the 'stimulus present' and 'stimulus omitted' conditions. These analyses revealed that the effect was driven by the fact that orientation-specific BOLD signals evoked by expected-but-omitted gratings had a different laminar profile in the two tasks (interaction between task and cortical layer, omission trials only: $F_{2,34} = 3.6$, $p = 0.039$; Fig. 3B, top panel). The laminar profile of stimulus-evoked activity, on the other hand, did not differ between the two tasks (interaction between task and cortical layer, stimulus present trials only: $F_{2,34} = 1.8$, $p = 0.18$; Fig. 3B, bottom panel). Following up on the interaction for the omission trials, we found that, during the orientation discrimination task runs, expected-but-omitted gratings evoked a significant orientation-specific BOLD signal in the deep ($t_{17} = 3.0$, $p = 0.0086$) but not in the middle ($t_{17} = 1.1$, $p = 0.28$; deep vs. middle: $t_{17} = 2.6$, $p = 0.020$) and superficial ($t_{17} = 0.1$, $p = 0.92$; deep vs. superficial: $t_{17} = 3.5$, $p = 0.0026$) layers. During the contrast discrimination task runs on the other hand, expected-but-omitted gratings did not evoke a significant orientation-specific BOLD signal in any layer (all $p > 0.4$). We now include these results in our manuscript (pp. 6-7), shedding a clearer light on the effects of task-relevance on the laminar profile of our expectation effects.

4. The omission condition tests the role of expectation that the authors hypothesize to involve feedback in deep layers, in contrast to prediction error that is hypothesized to involve superficial layers. This study tests the former but not the latter hypothesis. It would be stronger to show dissociation between processes engaging superficial vs. deep layers. Comparing expected and unexpected orientations would allow for testing for prediction error. I believe a previous study has tested this using the same behavioral paradigm but I couldn't see here any mention of trials with unexpected orientations. If the experimental design included both expected and unexpected orientations it would be helpful to compare the data across these conditions.

We certainly concur that it will be of great interest to compare the layer-specific effects of presenting expected vs. unexpected stimuli, i.e. prediction error. In fact, we are currently planning such a study, in follow-up of the current one. The reason for not including unexpected orientation was simply to obtain as many expected-but-omitted trials as possible. The current study is one of the first studies combining a fast event-related design with layer-specific fMRI, so we wanted to make sure we boosted the power of our study as much as possible. Given these encouraging results, we are excited about pursuing the prediction error question, and many others. We now mention this future direction in our Discussion (p. 10):

> "Therefore, we hypothesise that if, instead of omitting the predicted stimuli, one would present unexpected stimuli, this would lead to modulations in both deep and superficial layers. Future studies will be needed to test this hypothesis."

5. A previous study using the same paradigm showed that the expected orientation evokes a reduced response in V1 and this effect was not task specific. This is in contrast with the results presented here. How can the two studies be reconciled? It would be helpful to see the data from the two voxel sub-populations used to estimate BOLD differences between stimulus orientations. This analysis would help compare the results across studies.

The fact that the effects in the current study were task dependent was indeed surprising. As the reviewer notes, we did not report such a task dependence in a previous fMRI study (Kok et al., 2012) in which participants were presented with either expected gratings (as here) or unexpected gratings (rather than omissions, as here). In that study, the main comparison was between expected and unexpected gratings, while in the current study the interest lay in expected-but-omitted gratings. One, admittedly speculative, potential explanation is that the expectations during the contrast task, where orientation is task-irrelevant (unattended), evoked activity-silent synaptic plasticity that were not reflected in neural activity as measured in the BOLD signal (Rose et al., 2016; Wolff et al., 2017). When expectations were task-relevant on the other hand (i.e., during the orientation task), attention may boost the gain of expectation signals, promoting them from activity-silent to being reflected in neural firing. Previous studies have shown that activity-silent templates can be activated by TMS (Rose et al., 2016) or presenting a neural stimulus (Wolff et al., 2017). The interpretation is that this activity-silent synaptic plasticity 'shapes' how inputs (e.g. a TMS burst, top-down attentional gain, or a bottom-up stimulus) are processed. Therefore, both activity-silent and active expectation templates may modulate processing of subsequent stimuli, which may explain the absence of a task effect in our previous study comparing expected and unexpected stimuli (Kok et al., 2012). However, a simpler explanation may be that this previous study simply was not able to detect task modulations because it lacked laminar resolution; note that the task dependence here is expressed as an interaction between task and cortical layer. In sum, we currently cannot be sure what the precise reason for this discrepancy between studies, and future layer-specific research orthogonally manipulating attention and expectation validity will be needed to resolve this. These considerations are reflected on in our Discussion (pp. 12-13).

References

Albers AM, Kok P, Toni I, Dijkerman HC, De Lange FP (2013) Shared Representations for Working Memory and Mental Imagery in Early Visual Cortex. Current Biology 23:1427–1431.

Bastos AM, Lundqvist M, Waite AS, Kopell N, Miller EK (2020) Layer and rhythm specificity for predictive routing. bioRxiv:2020.01.27.921783.

Bastos AM, Usrey WM, Adams RA, Mangun GR, Fries P, Friston KJ (2012) Canonical Microcircuits for Predictive Coding. Neuron 76:695–711.

Bastos AM, Vezoli J, Bosman CA, Schoffelen J-M, Oostenveld R, Dowdall JR, De Weerd P, Kennedy H, Fries P (2015) Visual Areas Exert Feedforward and Feedback Influences through Distinct Frequency Channels. Neuron 85:390–401.

Bell AH, Summerfield C, Morin EL, Malecek NJ, Ungerleider LG (2016) Encoding of Stimulus Probability in Macaque Inferior Temporal Cortex. Current Biology 26:2280–2290.

Bijanzadeh M, Nurminen L, Merlin S, Clark AM, Angelucci A (2018) Distinct Laminar Processing of Local and Global Context in Primate Primary Visual Cortex. Neuron 100:259–274.

Cousineau D (2005) Confidence intervals in within-subject designs: A simpler solution to Loftus and Masson's method. Tutorials in Quantitative Methods for Psychology 1:42–45.

de Sousa AA, Sherwood CC, Schleicher A, Amunts K, MacLeod CE, Hof PR, Zilles K (2010) Comparative Cytoarchitectural Analyses of Striate and Extrastriate Areas in Hominoids. Cereb Cortex 20:966–981.

Fiser A, Mahringer D, Oyibo HK, Petersen AV, Leinweber M, Keller GB (2016) Experience-dependent spatial expectations in mouse visual cortex. Nat Neurosci 19:1658–1664.

Fracasso A, Petridou N, Dumoulin SO (2016) Systematic variation of population receptive field properties across cortical depth in human visual cortex. NeuroImage 139:427–438.

Harris KD, Mrsic-Flogel TD (2013) Cortical connectivity and sensory coding. Nature 503:51–58.

Harrison SA, Tong F (2009) Decoding reveals the contents of visual working memory in early visual areas. Nature 458:632–635.

Heinzle J, Koopmans PJ, den Ouden HEM, Raman S, Stephan KE (2016) A hemodynamic model for layered BOLD signals. NeuroImage 125:556–570.

Hinds OP, Rajendran N, Polimeni JR, Augustinack JC, Wiggins G, Wald LL, Diana Rosas H, Potthast A, Schwartz EL, Fischl B (2008) Accurate prediction of V1 location from cortical folds in a surface coordinate system. NeuroImage 39:1585–1599.

Hollander G de, Zwaag W van der, Qian C, Zhang P, Knapen T (2020) Ultra-high resolution fMRI reveals origins of feedforward and feedback activity within laminae of human ocular dominance columns. bioRxiv:2020.05.19.102186.

Hubel DH, Wiesel TN (1968) Receptive fields and functional architecture of monkey striate cortex. The Journal of physiology 195:215–243.

Huber L, Handwerker DA, Jangraw DC, Chen G, Hall A, Stüber C, Gonzalez-Castillo J, Ivanov D, Marrett S, Guidi M, Goense J, Poser BA, Bandettini PA (2017) High-Resolution CBV-fMRI Allows Mapping of Laminar Activity and Connectivity of Cortical Input and Output in Human M1. Neuron 96:1253-1263.e7.

Jehee JFM, Brady DK, Tong F (2011) Attention Improves Encoding of Task-Relevant Features in the Human Visual Cortex. Journal of Neuroscience 31:8210–8219.

Jordan R, Keller GB (2020) Opposing influence of top-down and bottom-up input on different types of excitatory layer 2/3 neurons in mouse visual cortex. bioRxiv:2020.03.25.008607.

Kaposvari P, Kumar S, Vogels R (2018) Statistical Learning Signals in Macaque Inferior Temporal Cortex. Cereb Cortex 28:250–266.

Keller GB, Mrsic-Flogel TD (2018) Predictive Processing: A Canonical Cortical Computation. Neuron 100:424–435.

Kok P, Bains LJ, van Mourik T, Norris DG, De Lange FP (2016) Selective Activation of the Deep Layers of the Human Primary Visual Cortex by Top-Down Feedback. Curr Biol 26:371–376.

Kok P, Jehee JFM, De Lange FP (2012) Less Is More: Expectation Sharpens Representations in the Primary Visual Cortex. Neuron 75:265–270.

Kumar S, Kaposvari P, Vogels R (2017) Encoding of Predictable and Unpredictable Stimuli by Inferior Temporal Cortical Neurons. Journal of Cognitive Neuroscience:1–10.

Larkum M (2013) A cellular mechanism for cortical associations: an organizing principle for the cerebral cortex. Trends in Neurosciences 36:141–151.

Lawrence SJ, Norris DG, De Lange FP (2019) Dissociable laminar profiles of concurrent bottom-up and top-down modulation in the human visual cortex Büchel C, Frank MJ, Büchel C, eds. eLife 8:e44422.

Lawrence SJD, Mourik T van, Kok P, Koopmans PJ, Norris DG, De Lange FP (2018) Laminar Organization of Working Memory Signals in Human Visual Cortex. Curr Biol 28:3435–3440.

Lee TS, Mumford D (2003) Hierarchical Bayesian inference in the visual cortex. Journal of the Optical Society of America A 20:1434.

Markuerkiaga I, Barth M, Norris DG (2016) A cortical vascular model for examining the specificity of the laminar BOLD signal. NeuroImage 132:491–498.

Markuerkiaga I, Marques JP, Gallagher TE, Norris DG (2020) Estimation of Laminar BOLD Activation Profiles using Deconvolution with a Physiological Point Spread Function. bioRxiv:2020.08.04.236190.

Meyer T, Olson CR (2011) Statistical learning of visual transitions in monkey inferotemporal cortex. PNAS 108:19401–19406.

Meyer T, Ramachandran S, Olson CR (2014) Statistical Learning of Serial Visual Transitions by Neurons in Monkey Inferotemporal Cortex. Journal of Neuroscience 34:9332–9337.

Morey RD (2005) Confidence intervals from normalized data: A correction to Cousineau. Tutorials in Quantitative Methods for Psychology 4:61–64.

Raizada RDS, Grossberg S (2003) Towards a Theory of the Laminar Architecture of Cerebral Cortex: Computational Clues from the Visual System. Cereb Cortex 13:100–113.

Rockland KS, Pandya DN (1979) Laminar origins and terminations of cortical connections of the occipital lobe in the rhesus monkey. Brain Research 179:3–20.

Rose NS, LaRocque JJ, Riggall AC, Gosseries O, Starrett MJ, Meyering EE, Postle BR (2016) Reactivation of latent working memories with transcranial magnetic stimulation. Science 354:1136–1139.

Scheeringa R, Koopmans PJ, Mourik T van, Jensen O, Norris DG (2016) The relationship between oscillatory EEG activity and the laminar-specific BOLD signal. PNAS:201522577.

Schwiedrzik CM, Freiwald WA (2017) High-Level Prediction Signals in a Low-Level Area of the Macaque Face-Processing Hierarchy. Neuron 96:89-97.e4.

Self MW, van Kerkoerle T, Goebel R, Roelfsema PR (2019) Benchmarking laminar fMRI: Neuronal spiking and synaptic activity during top-down and bottom-up processing in the different layers of cortex. NeuroImage 197:806–817.

Serences JT, Boynton GM (2007) Feature-Based Attentional Modulations in the Absence of Direct Visual Stimulation. Neuron 55:301–312.

Serences JT, Ester EF, Vogel EK, Awh E (2009) Stimulus-Specific Delay Activity in Human Primary Visual Cortex. Psychological Science 20:207–214.

Spratling MW (2008) Reconciling Predictive Coding and Biased Competition Models of Cortical Function. Frontiers in Computational Neuroscience 2 Available at: http://journal.frontiersin.org/article/10.3389/neuro.10.004.2008/abstract [Accessed April 7, 2016].

Treue S, Trujillo JCM (1999) Feature-based attention influences motion processing gain in macaque visual cortex. Nature 399:575–579.

Van Kerkoerle T, Self MW, Roelfsema PR (2017) Layer-specificity in the effects of attention and working memory on activity in primary visual cortex. Nature Communications 8:13804.

Van Mourik T, Koopmans PJ, Norris DG (2019) Improved cortical boundary registration for locally distorted fMRI scans. PLOS ONE 14:e0223440.

Vinken K, Vogels R, Beeck HO de (2017) Recent Visual Experience Shapes Visual Processing in Rats through Stimulus-Specific Adaptation and Response Enhancement. Current Biology 27:914–919.

Wolff MJ, Jochim J, Akyürek EG, Stokes MG (2017) Dynamic hidden states underlying working-memory-guided behavior. Nat Neurosci 20:864–871.

Zmarz P, Keller GB (2016) Mismatch Receptive Fields in Mouse Visual Cortex. Neuron 92:766–772.
