# Supplementary material for: Prior expectations evoke stimulus-specific activity in the deep layers of the primary visual cortex
Source: PLoS Biol. 2020 Dec 7;18(12):e3001023. doi: 10.1371/journal.pbio.3001023 (PMC7746273; doi:10.1371/journal.pbio.3001023)
Supplement: Attachment — Submitted filename: Response_to_reviewers.docx [file pbio.3001023.s009.docx]

We would like to thank the reviewers for their positive assessment of our revised manuscript. We have revised the manuscript to address the final remaining issues, as detailed below.

**Reviewer 1**

*1. Thank you for adding the additional details about data handling -- the revised manuscript gives the reader a good sense of the data. The illustration of the anatomical landmarks and regions of interest (selected voxels) in Fig. 2 is very helpful. The legend should probably include a note that the red and yellow lines are the pial and wm surfaces of the GM after warping via RBR to the functional data. It's clear from this figure that RBR struggled with the contrast in the EPI and sometimes underestimated the cortical thickness, but I think this is a challenge confronted by the entire field, and the additional analyses provided in the main ms and the supplemental figures provide reasonable confidence that the unavoidable imperfections in registration/GM definition did not produce systematic bias.*

Thank you for this recommendation, we have now added this information about the red and yellow lines in Figure 2 to the figure legend:

“Overlaid red and yellow lines indicate pial and white matter boundaries, respectively, as determined by registering anatomical boundaries to the mean functional image using recursive boundary registration (RBR).”

*2. The additional figure created to support the review process, which shows the negative BOLD response to the omitted stimuli, helps a great deal with interpretation and understanding how analysis was done. I think including it in the supplemental material, along with the excellent explanation provided for the review, is a good idea.*

We are glad that the reviewer found that this additional figure has made our results easier to interpret, and we now include it as Figure S1, including the explanation of the results in the figure legend.

**Reviewer 2**

*In my review of an earlier version of the manuscript, I had raised concerns that the presentation was too abbreviated to judge the merits of the experiment based on the data. My comments were echoed by two other reviewers. For this revision of the manuscript, the authors went through substantial amount of effort to address my main concern (that presentation was too condensed for reviewers / readers). I think the work that went into the revision has made it a much better paper.

R1 and R3 raised additional, more detailed methodological questions, which are carefully addressed in this revision. As a result, the manuscript now lays out a much clearer and easier to understand logic. Crucially, it includes much more detail about the dataset and intermediate processing steps, which previously had to be inferred from related publications.

At this point, I have no further concerns.*

We are glad to note that the reviewer found that our revision addressed all of their concerns.

**Reviewer 3**

*1. The authors have responded to my initial comments satisfactorily. However, I do have a concern about one issue raised by Reviewer 1, point 4 regarding the voxel selection (and I apologize for not raising this point in the initial review). The authors new analysis rule out a bias due to unequal sampling of different laminae, but there is another potential issue which is to do with the spatial distribution of voxels on the cortical surface. Assuming Fig 2A is representative, it is clear that only a tiny fraction of activated voxels are included among the selected voxels, and these appear to be scattered widely across the V1 ROI. Given that the conclusions hinge on the ability to assign differences in responses solely to laminar position, the authors need to demonstrate that the voxels from each layer are distributed randomly across the surface and not clustered in some systematic way (e.g. by eccentricity or angular position). This could be done for instance by plotting the
voxels on the cortical surface and labeling them by laminar position and check this plot for clustering (supported by a suitable statistical procedure). Alternatively, they could demonstrate that any variations in response strength along the cortical surface across all layers could not account for the effects seen. Or, better still, they could redo the laminar analysis including increasingly large numbers of voxels and show that the results remained qualitatively similar. There is considerable evidence that orientation preference in V1 is driven by large-scale spatial patterns rather than columnar sampling (this is actually evident in Fig 2A, where voxels tuned to the different orientations are spatially clustered) so lowering the threshold for voxel inclusion should not be expected to have dramatic effects on orientation tuning of the voxels. As Reviewer 1 points out, the voxel selection method is very restrictive and it needs to be shown that the results are not influenced
by this procedure.*

We thank the reviewer for these suggestions. We have implemented the reviewer’s preferred solution, i.e., redoing the laminar analysis with increasing numbers of voxels and demonstrating that the results remain qualitatively similar. We have included this analysis in the manuscript, and present the results in a new Figure S4 (reproduced below for convenience). As can be seen, the expected-but-omitted gratings (Fig. S4A) only ever evoked a reliable orientations-specific BOLD response in the deep layers of V1, while for presented gratings the response was always highest in the superficial layers (Fig. S4B).

“The main results were based on the 500 V1 voxels most selective for 45° and 135° oriented gratings, respectively. In order to establish whether the results depended on the exact number of voxels selected, we conducted a control analyses which quantified the orientation-selective BOLD response evoked by presented and expected-but-omitted gratings as a function of the number of selected voxels, ranging from 100 to 1000 voxels per orientation. Even though the orientation-selective BOLD response overall decreased as more less-selective voxels were added, the main results were qualitatively similar over the range of selected voxels (Figure S4), establishing that the layer-specific effects of expectation did not depend on the exact number of selected voxels.”

**Figure S4. Layer-specific BOLD response in V1 for presented and expected as a function of the number of selected voxels. (A)** Orientation-specific BOLD response to expected-but-omitted gratings in the different layers of V1, averaged over tasks. **(B)** Orientation-specific BOLD response to presented gratings in the different layers of V1, averaged over tasks. Error bars indicate within-subject SEM.

**Reviewer 4**

*1. The authors have addressed my comments related to data analyses and statistics and I believe the results presented in the revised manuscript are stronger. The authors explain the difference between expectation and attention/working memory. It would be helpful if the authors discussed the role of mental imagery and how it relates to expectation. Recent work suggests that mental imagery relates to BOLD in deeper layers, similar to the results for expectation presented by the authors.*

We thank the reviewer for this suggestion, and have now included a paragraph on this topic in the Discussion:

“Recent work has revealed that mental imagery can also induce stimulus-specific signals selectively in the deep layers of V1 (Bergmann et al., 2019). This suggests that perhaps the crucial factor determining the involvement of the superficial layers in top-down modulations lies in whether they involve keeping a presented stimulus online (as in most working memory tasks) or generating a stimulus presentation *de novo* (as in mental imagery tasks and in the omission trials in the current study). Further research will be needed to address this question, as well as determine whether or not mental imagery and expectation involve distinct neural mechanisms (Pearson and Westbrook, 2015).”

**References**

Bergmann J, Morgan AT, Muckli L (2019) Two distinct feedback codes in V1 for ‘real’ and ‘imaginary’ internal experiences. bioRxiv:664870.

Pearson J, Westbrook F (2015) Phantom perception: voluntary and involuntary nonretinal vision. Trends in Cognitive Sciences 19:278–284.
