# Supplementary material for: Prior expectations evoke stimulus-specific activity in the deep layers of the primary visual cortex
Source: PLoS Biol. 2020 Dec 7;18(12):e3001023. doi: 10.1371/journal.pbio.3001023 (PMC7746273; doi:10.1371/journal.pbio.3001023)
Supplement: Attachment — Submitted filename: Response_to_reviewers.docx [file pbio.3001023.s010.docx]

We would like to thank the reviewers for their positive assessment of our revised manuscript. We were glad to note that there were no further outstanding comments to address.
